# Mutation-specific non-canonical pathway of PTEN as a distinct therapeutic target for glioblastoma

**DOI:** 10.1038/s41419-021-03657-0

**Published:** 2021-04-07

**Authors:** Seung Won Choi, Yeri Lee, Kayoung Shin, Harim Koo, Donggeon Kim, Jason K. Sa, Hee Jin Cho, Hye-mi Shin, Se Jeong Lee, Hyunho Kim, Seok Chung, Jihye Shin, Cheolju Lee, Do-Hyun Nam

**Affiliations:** 1 Department of Neurosurgery, Sungkyunkwan University School of Medicine, Samsung Medical Center, Seoul, Republic of Korea; 2 Institute for Refractory Cancer Research, Samsung Medical Center, Seoul, Republic of Korea; 3 Department of Health Sciences and Technology, Samsung Advanced Institute for Health Sciences and Technology (SAIHST), Sungkyunkwan University, Seoul, Republic of Korea; 4 BK21 Graduate Program, Department of Biomedical Sciences, Korea University College of Medicine, Seoul, Republic of Korea; 5 Research Institute for Future Medicine, Samsung Medical Center, Seoul, Republic of Korea; 6 Department of Biomedical Convergence Science and Technology, Kyungpook National University, Daegu, Republic of Korea; 7 Department of Anatomy, Korea University College of Medicine, Seoul, Republic of Korea; 8 School of Mechanical Engineering, College of Engineering, Korea University, Seoul, Republic of Korea; 9 KU-KIST Graduate School of Converging Science and Technology, Korea University, Seoul, Republic of Korea; 10 Center for Theragnosis, Korea Institute of Science and Technology, Seoul, Republic of Korea; 11 Division of Bio-Medical Science & Technology, KIST School, Korea University of Science and Technology, Seoul, Republic of Korea

**Keywords:** Cancer, Oncogenes

## Abstract

*PTEN* is one of the most frequently altered tumor suppressor genes in malignant tumors. The dominant-negative effect of *PTEN* alteration suggests that the aberrant function of PTEN mutation might be more disastrous than deletion, the most frequent genomic event in glioblastoma (GBM). This study aimed to understand the functional properties of various *PTEN* missense mutations and to investigate their clinical relevance. The genomic landscape of *PTEN* alteration was analyzed using the Samsung Medical Center GBM cohort and validated via The Cancer Genome Atlas dataset. Several hotspot mutations were identified, and their subcellular distributions and phenotypes were evaluated. We established a library of cancer cell lines that overexpress these mutant proteins using the U87MG and patient-derived cell models lacking functional *PTEN*. *PTEN* mutations were categorized into two major subsets: missense mutations in the phosphatase domain and truncal mutations in the C2 domain. We determined the subcellular compartmentalization of four mutant proteins (H93Y, C124S, R130Q, and R173C) from the former group and found that they had distinct localizations; those associated with invasive phenotypes (‘edge mutations’) localized to the cell periphery, while the R173C mutant localized to the nucleus. Invasive phenotypes derived from edge substitutions were unaffected by an anti-PI3K/Akt agent but were disrupted by microtubule inhibitors. *PTEN* mutations exhibit distinct functional properties regarding their subcellular localization. Further, some missense mutations (‘edge mutations’) in the phosphatase domain caused enhanced invasiveness associated with dysfunctional cytoskeletal assembly, thus suggesting it to be a potent therapeutic target.

## Introduction

Deletion in chromosome 10 is a hallmark of glioblastoma (GBM) regarding copy-number variations. Thus, *PTEN* deletion, given its location in 10q23.31, has been considered the primary oncogenic driver in the development of GBM^1^. Studies using The Cancer Genome Atlas (TGCA) have shown frequent *PTEN* mutations as well as deletions in GBM cohorts. Multiple studies have documented the dominant-negative effect of *PTEN* mutation, which suggests that aberrant gain of function attributed to *PTEN* mutation might be more fatal than the mechanical loss of function driven by *PTEN* deletion in the perspective of malignant potential^2^. This finding also suggests the possibility of mutation-specific therapeutic vulnerability of *PTEN*. Several hotspot *PTEN* mutations have been well recognized; however, their specific functions as well as phenotypical and clinical relevance remain elusive.

*PTEN* is a well-known tumor suppressor gene which is frequently lost or mutated in hereditary and sporadic cancers^3,4^. *PTEN* encodes a 403-residue protein with four major domains: a PtdIns^4,5^-P2 binding domain (PBD), a phosphatase domain, a C2 domain, and a C2 tail^5^, which contribute to distinct functional characteristics. The catalytic function of the phosphatase domain is usually emphasized and has dual specificities for phosphoproteins and phospholipids^6–8^. As a protein phosphatase, PTEN has been shown to dephosphorylate phosphopeptides in vitro^9^. Its phosphoprotein targets include the focal adhesion kinase c-SRC and PTEN itself^8,10,11^. PTEN catalyzes the hydrolysis of the second messenger PtdIns^3–5^-P3 (PIP3), which counteracts the activation of the PI3K/Akt pathway, thus regulating cellular growth, proliferation, and metabolism^6,12,13^. Although tumor suppressor genes such as *PTEN* per se may not be considered optimal therapeutic targets, their loss of function via various canonical pathways constitutes cellular dependency that could be exploited therapeutically. Therefore, in-depth understanding of the molecular mechanism behind each *PTEN* mutation could present novel therapeutic opportunities in GBM treatment.

In this study, we describe the mutational landscape of *PTEN* in GBM patients and experimentally validate the functional properties of several hotspot substitutions that are found in the phosphatase domain of PTEN. Through combining bioinformatics analyses, we further categorized *PTEN* mutations and showed their phenotypical relevance. We also illustrated the clinical significance of *PTEN* mutations by analyzing the radiographic failure patterns observed in GBM patients.

## Results

### Genomic landscape of *PTEN* in glioblastoma

**Fig. 1:**
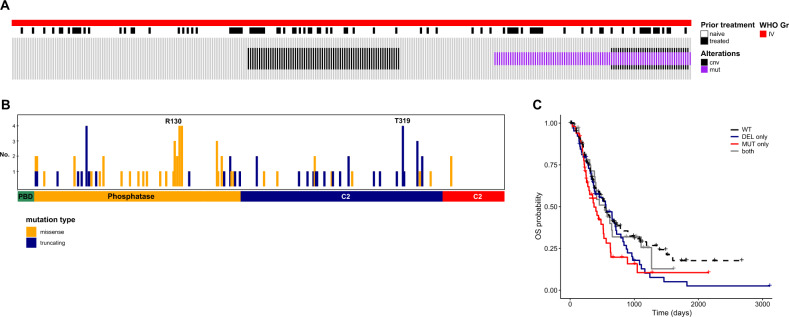
Genomic landscape of *PTEN* in glioblastoma patients. **A** Genomic landscape of *PTEN* in glioblastoma (GBM) in the study cohort. **B** Mutation profiles of *PTEN* alterations in GBM tumors. Statistically significant association between mutation type and PTEN domain was identified; missense mutations were dominant in the phosphatase domain, while truncating mutations were prevalent in the C2 domain (*p*-value < 0.001, Fisher’s exact test). **C** Survival outcome of GBM patients stratified by *PTEN* alteration status. There was no statistically significant difference in prognosis as a function of the alteration status of *PTEN* (*p*-value = 0.1, Log-rank test). Survival analysis was performed using Kaplan–Meier method and log-rank test was used to statistically compare the curves. cnv, copy-number variation; mut, mutation; no., number; OS, overall survival; WT, wild-type; DEL-only, deletion only; MUT-only, mutation only; both, both mutation and deletion alleles.

To explore the distribution of *PTEN* alterations in glioblastoma (GBM) patients, we analyzed the genomic profiles of 303 tumors derived from 252 patients of Samsung Medical Center (SMC) GBM cohort (Fig. 1A). As expected, most GBM tumors were IDH1-wild-type (WT) (93.1%, 282/303), and *PTEN* mutation was exclusively found in these IDH1-WT tumors (*P*-value < 0.001, Fisher’s exact test) (Supplementary Fig. S1). *PTEN* mutations were identified in almost a third of the cases (29.0%, 88/303), and *PTEN* deletion was confirmed in 39.6% of tumors (103/260). Among patients with available mutation profiles and copy-number variation data, only 13.5% (35/260) exhibited concurrent *PTEN* deletion and mutation.

We further analyzed *PTEN* mutations according to mutation type and location. Majority of the mutations were located within the phosphatase and C2 domains (Fig. 1B). Interestingly, missense mutations were dominant in the phosphatase domain, while truncating mutations were more prevalent in the C2 domain. This predominance was also evident in the TCGA dataset (Supplementary Fig. S2).

To explore the clinical implication of *PTEN* mutations, we analyzed the survival outcomes of patients, stratified by *PTEN* alteration status. Regarding the dominant-negative effect of *PTEN* mutation^2^, patients were categorized as follows; mutation only, deletion only, or with both mutation and deletion alleles. As illustrated in Fig. 1C, there was no statistically significant difference in prognosis as a function of the alteration status of *PTEN*, however, patients with *PTEN* mutation only showed the shortest median overall survival (OS) compared to those with deletion only or both alterations (median OS, 286, 384, 408 and 437 days for patients with mutation only, deletion only, both alteration and wild type (WT), respectively). These findings suggested that missense mutations causing aberrant gain of function in *PTEN* might have more profound clinical implication than the loss of function by deletion. This finding was also concordant with survival outcomes of the TCGA cohort (Supplementary Fig. S3).

### Establishment of cancer cell lines overexpressing mutant PTEN proteins

As described above, majority of *PTEN* mutations were missense mutations within the phosphatase domain or truncating mutations within the C2 domain. The molecular functions of PTEN are diverse, complex, and primarily associated with the catalytic activity of the phosphatase domain^14^. Therefore, we focused on exploring the functional properties of missense mutations in this domain.

From analyzing the literature review and our study cohort, several *PTEN* hotspot mutations were identified: D24N, H93Y, C124S, R130Q, G132D, R173C, and K289E. Of these, five missense mutations (D24N, H93Y, R130Q, G132D, and R173C) are known to be maintained throughout the temporal evolution of GBMs^15^ while the other two (C124S and K289E) are well-known mutations in previous *PTEN* studies^9,16^. The catalytically inactive C124S substitution is a representative loss-of-function substitution, whereas the K289E substitution, which is located within the C2 domain, retains WT phosphatase activity.

**Fig. 2:**
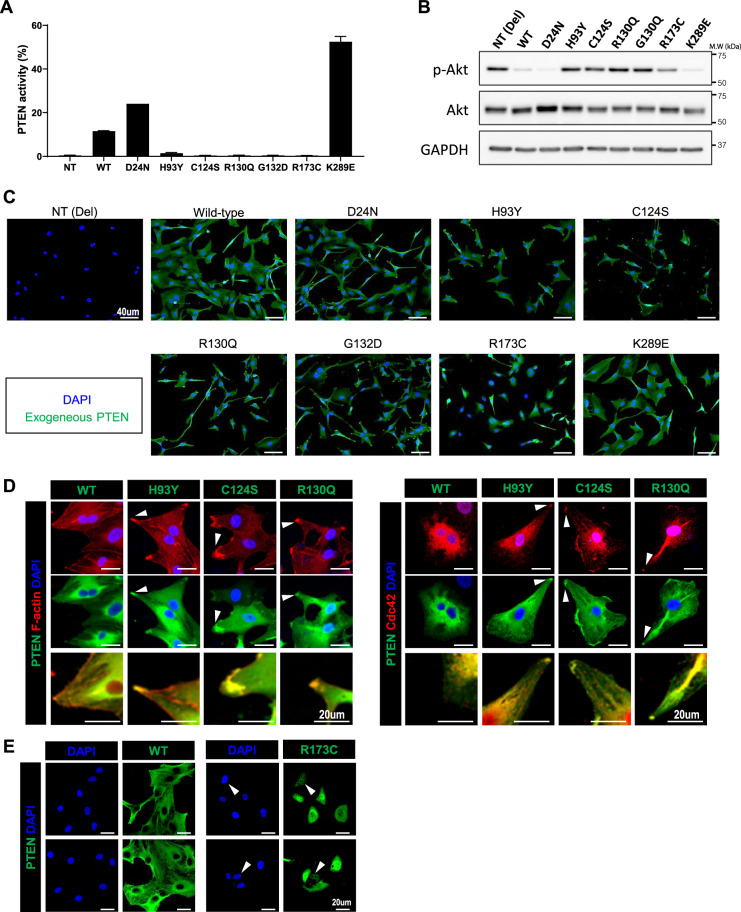
Distinct subcellular compartmentalization of PTEN mutants. We established cell lines overexpressing different PTEN mutants of interest using U87MG cells. **A** PTEN activity was measured by converted ratio of PI-^4,5^-P2 from supplemented PI-^3–5^-P3 by PTEN protein in U87MG cell lines with exogenous PTEN mutants. Data are shown as the means of triplicates of experiments ± s.d. **B** p-Akt activity of U87MG cell lines expressing wild-type PTEN and PTEN mutants. **C** Subcellular compartmentalization differed by *PTEN* mutations—some mutations (H93Y, C124S, and R130Q) with cytoplasmic distribution exhibited peculiar localization at cell periphery, while another mutation (R173C) showed nuclear localization. **D** PTEN mutants located in the cytoplasmic compartment co-localized with markers of the leading edge in migrating cells, Cdc42 and F-actin (white arrow). High-resolution images depicting the co-staining of PTEN and F-actin are presented at the bottommost row. **E** Nuclear compartmentalization of the R173C PTEN mutant (white arrow). WT, wild-type; NT, null-type; Del, deletion; s.d: standard deviation.

To address the functional implication of these mutations, we established PTEN mutants-overexpressing cell lines using *PTEN*-null cells. U87MG cell line has in-frame deletion within exon 3 of *PTEN*, and usually used as a negative control for *PTEN* functional studies^17^. Intact PTEN phosphatase activity, measured by the converted ratio of PI-^4,5^-P2, was confirmed in the WT, D24N, and K289E overexpressing U87MG cell lines, whereas cells overexpressing other PTEN mutants exhibit no phosphatase activity (Fig. 2A). Accordingly, increased Akt phosphorylation was observed in U87MG cell lines expressing the phosphatase domain mutants (Fig. 2B). *PTEN*-null PDCs (P089 and P090) were also used to establish the PTEN-mutant-overexpressing cells. The absence of phosphatase activity in PDCs with overexpressing the PTEN mutants of the interest was confirmed (Supplementary Fig. S4).

### Subcellular localizations of PTEN mutants varied by mutation type

The molecular mechanism behind the tumor-suppressive function of *PTEN* can differ based on subcellular compartmentalization^16,18–21^. To comprehend the functional implication of *PTEN* mutations in the present study, we examined the subcellular localization of PTEN mutants using U87MG cell lines, infected by lentivirus carrying PTEN mutant DNA (H93Y, D24N, R130Q, G132D, C124S, or R173C).

We observed that subcellular compartmentalization varied by *PTEN* substitutions, even for mutations derived from the same phosphatase domain. Most PTEN mutants were located within the cytoplasm, while R173C was exclusively found in the nucleus (Fig. 2C). Interestingly, the H93Y, C124S, and R130Q substitutions were particularly found at the cell periphery (Fig. 2C).

PTEN catalyzes the conversion of PI-^3–5^-P3 into PI-^4,5^-P2, with these phosphoinositides playing a key role in defining the polarity of migrating cells^22^. To examine the effect of *PTEN* mutation on cellular polarity, immunofluorescence staining study was performed. As shown in Fig. 2D, F-actin and Cdc42 were co-localized with overexpressing PTEN mutants in U87MG cells, which implied that these PTEN mutants accumulated at the front of cells, regarding their polarity. In contrast, the PTEN R173C mutant was solely located within the nucleus (Fig. 2C, E). We have termed the cytoplasmic mutations as “edge mutations” (H93Y, C124S, and R130Q) and R173C as “nuclear mutation”.

### CLUMP analysis of *PTEN* mutation exhibited distinct categorization

Substitutions in the same phosphatase domain exhibited varied subcellular compartmentalization, which could attribute to their distinct functions; such a finding was contrary to the traditional notion of the similar function of mutations derived from the same domain.

**Fig. 3:**
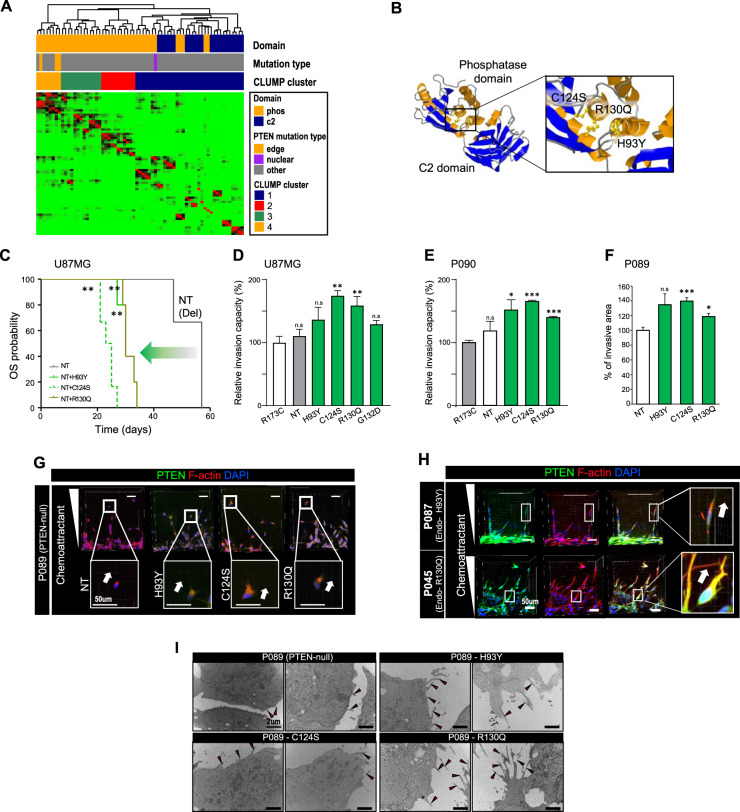
Invasive phenotype of PTEN edge mutants. U87MG cells and *PTEN*-null PDCs (P089 and P090) were used to overexpress PTEN edge mutants. PDCs with endogenous *PTEN* edge mutation were also used for validation (P045 with R130Q mutation; P087 with H93Y mutation). **A** CLUMP analysis of *PTEN* mutations identified in SMC and TCGA cohorts. A total of 68 residues were finally selected for CLUMP analysis and CLUMP analysis revealed four distinct subgroups of *PTEN* mutations. According to CLUMP analysis, *PTEN* mutations within the phosphatase domain were further divided into distinct subgroups, which correlated with the 3D structure of PTEN protein**. B** 3D crystal structure of the PTEN protein: all residues changed by *PTEN* edge mutations (H93, C124, and R130) are located within the same pocket of the phosphatase domain. **C** Comparison of survival outcomes of orthotopic xenografts established by U87MG cells with distinct PTEN mutants. The mice with edge mutations showed poor survival outcome compared to PTEN-null mice (*P*-value <0.001, Log-rank test). Three *PTEN*-null xenografts were used as control group, while six PTEN-mutant xenografts were made per each mutation of interest. **D**–**F** In vitro invasion assay to assess the enhanced invasion capacity of *PTEN* edge mutation compared to nuclear mutation. Data are shown as the means of triplicates of experiments ± s.d. The *P-value* was calculated by two-sided *t* test. **D** In vitro trans-well invasion assay using U87MG cells with exogenous mutants. All tumor cells with edge mutants exhibited significantly increased invasion capacity compared to tumors with nuclear mutants, except H93Y (*p*-value=0.076, 0.005, and 0.008 for H93Y, C124S, and R130Q, respectively, two-sided *t* test). Their enhanced invasion capacity was also significantly superior to *PTEN*-null U87MG cells (*p*-value=0.203, 0.001, and 0.015 for H93Y, C124S, and R130Q, respectively, two-sided *t* test). **E** in vitro 3D sphenoid invasion assay using *PTEN*-null PDCs (P090) with exogenous mutants. All PDCs (P090) with edge mutants exhibited significantly enhanced invasion capacity compared to PDCs with nuclear mutation (*p*-value=0.028, <0.001, and <0.001 for H93Y, C124S, and R130Q, respectively, two-sided *t* test). **F** In vitro microfluid assay to measure the development of invasion in response to chemotactic stimuli in *PTEN*-null PDCs (P089) with exogenous mutants. PDCs expressing edge mutants showed a more invasive phenotype than *PTEN*-null PDCs (*p*-value=0.135, <0.001, and 0.023 for H93Y, C124S, and R130Q, respectively, two-sided *t* test). **G**, **H** Co-localization of F-actin and PTEN edge mutants at the cell periphery of motile PDCs upon chemotactic stimuli. **I** Development of cellular projections (black arrow) of PDCs (P089) with exogenous *PTEN* edge mutations. NT, null-type; Del, deletion.

CLUMP (clustering of mutations in protein structure) is a computational method to predict the significance of mutation in a given 3D structure^23^. To account for the inconsistency found in the analysis of the subcellular location of phosphatase domain mutations, we hypothesized that each residue’s implication in a 3D conformal structure might be more relevant to predict the practical function of each residue. Using CLUMP, we presumed the potential functional subgroups among different *PTEN* mutations found in SMC and TCGA cohorts. All edge mutations were clustered together (CLUMP cluster 4), while the nuclear mutation was clustered separately into a different subgroup (CLUMP cluster 1), which was rather close to mutations that are found in the C2 domain (Fig. 3A). In addition, we observed that mutations of different CLUMP-defined clusters exhibited distinct subcellular localization. PTEN mutants from G129E (CLUMP cluster 4) exhibited cytoplasmic localization while those of Y177C (CLUMP cluster 1) showed nuclear compartmentalization, which supporting the functional relevance of CLUMP-defined clusters (Supplementary Fig. S5).

The crystal structure of the PTEN protein showed that all residues in edge mutations located within the same pocket of the phosphatase domain in the crystal structure, where PI-^3–5^-P3 binds (Fig. 3B). This finding signifies the distinct functions of edge and nuclear mutations, as illustrated by their differential subcellular compartmentalization.

### Edge mutants have invasive phenotypes

PTEN edge mutants (H93Y, C124S, and R130Q) were found in the cytoplasmic compartment, especially at the fronts of cells. The development of leading edges is associated with invasiveness and activation of the PI3K/Akt signaling pathway^24,25^. Moreover, in vivo experiments revealed that mice with edge mutations showed poor survival outcome compared to *PTEN*-null mice, and this finding prompted us to examine the phenotypic characteristics of these mutations (Fig. 3C). Interestingly, xenograft models with nuclear mutation (R173C) exhibited better prognosis than *PTEN*-null models (*p*-value=0.033, log-rank test), which indirectly supported the more pronounced hazardous effect of edge mutation on the prognosis (Supplementary Fig. S6).

To examine the phenotypical characteristics of edge mutations, we established the U87MG cells with exogenous PTEN mutants. According to in vitro trans-well invasion assay, all tumor cells with edge mutants exhibited significantly increased invasion capacity compared to tumors with nuclear mutants, except H93Y (Fig. 3D and Supplementary Fig. S7). Their enhanced invasion capacity was also significantly superior to *PTEN*-null U87MG cells. This finding was further validated by in vitro 3D spheroid invasion assay using *PTEN*-null PDCs (P090) with exogenous mutants (Fig. 3E). In vitro microfluid assay also confirmed that PDCs with edge mutation exhibited a more invasive phenotype than *PTEN*-null PDC (Fig. 3F and Supplementary Fig. S8).

Tumors with H93Y and G132D mutations showed increased invasive capacity compared to tumors with *PTEN*-null or nuclear mutation, however, failed to exhibit statistically significant difference. We assumed that variability in data might be one of the reasons behind this statistical insignificance despite their tendency toward enhanced invasion.

Local elevation of PI-^3–5^-P3 is associated with actin polymerization, promoting the development of the leading edge in motile cells^26–28^. Its spatial distribution is regulated by spatial modulation of PTEN and PI3K. We found that PTEN mutants were localized at the leading edge of chemotaxing cells. PDCs (P089) with exogenous edge mutations showed co-localization of PTEN mutants with F-actin at the leading edge in compliance with the chemotactic slope (Fig. 3G and Supplementary Fig. S9). PDCs with endogenous edge mutations (P087(H93Y) and P045(R130Q)) also showed similar spatial patterns of PTEN accumulation (Fig. 3H and Supplementary Fig. S10).

Additionally, we examined the development of cellular projections of edge mutations using a transient electron microscope (TEM)^29^. Protrusion formation (e.g. filopodia and lamellipodia) is closely related to the invasive phenotype and migration in various cancers^30,31^. As shown in Fig. 3I, abundant filopodia were present in PDCs expressing edge mutants, compared to *PTEN*-null PDCs. Additionally, their filopodia were longer than those of control cells. These results suggest that invasive mutant-harboring GBM cells exhibited more infiltrative characteristics, such as filopodia, and lamellipodia.

### Edge-mutant phenotypes are disrupted by microtubule inhibitors, but not by an PI3K/Akt inhibitor

We confirmed that *PTEN* edge mutations induced the invasive phenotype through in vitro experiments. All these edge mutations are located within the same pocket of the phosphatase domain of PTEN, hence suggesting their similar function in the context of the PTEN catalytic cascade. PI3K/Akt signaling is downstream of PTEN, associated with invasion and treatment resistance in cancer^32^. Several anti-PI3K/Akt signaling drugs have been developed for clinical use^33–35^; BKM120, a pan-class I PI3K inhibitor, is one of the most advanced agents readily crossing the blood-brain barrier^36^.

**Fig. 4:**
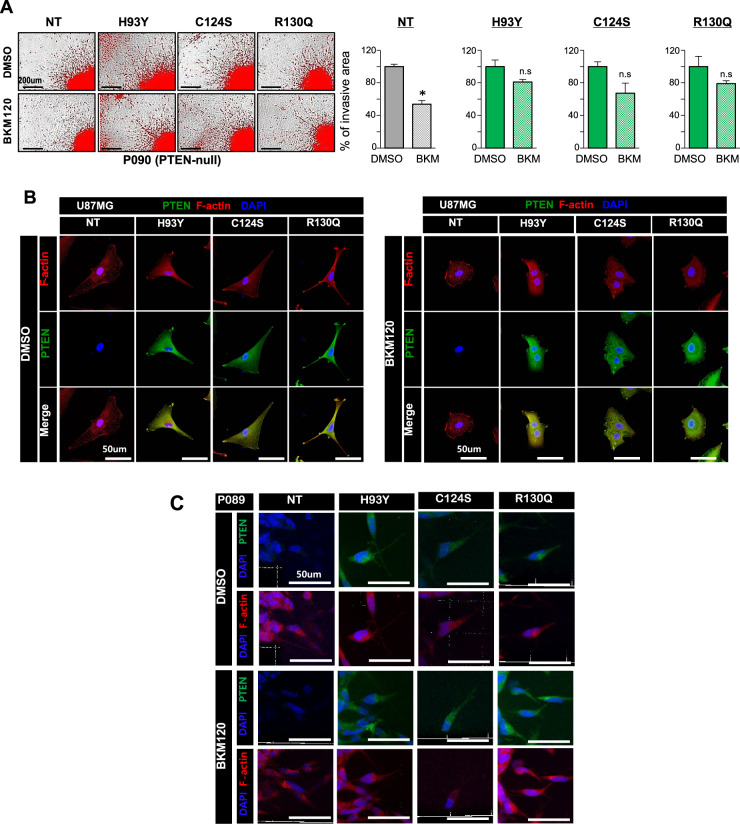
Invasive phenotypes of PTEN edge mutants are not altered by BKM120, a PI3K/Akt inhibitor. **A** Development of invasive branches in *PTEN*-null PDCs (P090) overexpressing *PTEN* edge mutation was not altered by BKM120, a pan-PI3K inhibitor (*p*-value = <0.001, 0.091, 0.071, and 0.193 for NT, H93Y, C124S, and R130Q, two-sided *t* test). Data are shown as the means of four experimental replicates ± s.d. **B**, **C** Persistence of PTEN edge mutants at the cellular edge of U87MG cells and *PTEN*-null PDCs (P089) with exogenous edge mutation following BKM120 treatment. NT, null-type; del, deletion; conc., concentration.

*PTEN*-null PDCs (P090) with exogenous mutants were treated with BKM120 to inhibit the PI3K/Akt signaling pathway. Surprisingly, invasive phenotypes were not significantly diminished by BKM120, compared to *PTEN*-null PDCs (Fig. 4A). Edge mutants were still present at leading edges while retaining their invasive branches in U87MG cells with edge mutants (Fig. 4B). Persistent co-localization of F-actin and edge mutants in P089 with edge mutants following BKM120 treatment further supports the therapeutic resistance against BKM120 in these cells (Fig. 4C). These data suggested that the invasive phenotype of edge mutations is not dependent on the PI3K/Akt signaling pathway.

**Fig. 5:**
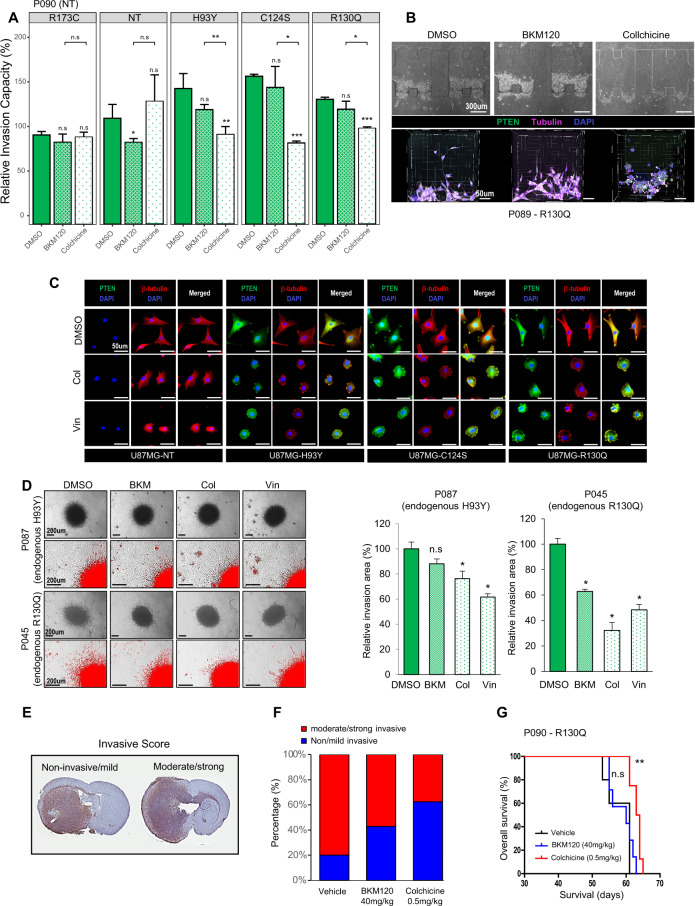
A microtubule inhibitor disrupts invasive phenotypes of PTEN edge mutants in vitro and in vivo. **A** Decrease in invasion capacity following microtubule inhibitor treatment (Colchicine) in *PTEN*-null PDCs (P090) overexpressing PTEN edge mutants. Data are shown as the means of triplicates of experiments ± s.d. PDCs expressing edge mutants exhibited significantly fewer invasive cellular projections following colchicine treatment (*p*-value=0.009, <0.001, and <0.001 for H93Y, C124S, and R130Q, one-sided *t* test). **B** Decrease in invasion following microtubule inhibitor treatment in *PTEN*-null PDCs with exogenous edge mutation (P089-R130Q) observed in microfluid assay; morphological changes captured during therapy are presented below, which reflecting less invasive property. **C** Development of tubulin aggresomes after microtubule inhibitor treatment in U87MG cells overexpressing *PTEN* edge mutations. **D** Decreased invasive branches of PDCs with endogenous edge mutation following microtubule inhibitor treatment. Data are shown as the means of four experimental replicates ± s.d. PDCs with endogenous edge mutations exhibited significantly decreased invasion following colchicine treatment than BKM120 (*p*-value=0.114, 0.029, and 0.029 for BKM120, Col, and Vin, respectively (P087); *p*-value=0.029, 0.029, and 0.029 for BKM120, Col, and Vin, respectively (P045), two-sided Wilcoxon test). **E**–**G** Intracranial xenograft model was established using *PTEN*-null PDCs with exogenous edge mutants (P090-R130Q); Five xenografts were treated by vehicle, while seven and eight mice were treated by BKM120 and Colchicine, respectively. **E** Invasive scoring system to evaluate the severity of invasion in vivo. We redefined the original invasion scoring system into binary classification, “non-invasive/mild” versus “moderate/strong”. The extent of tumor cell transmission via corpus callosum is the main criteria to distinguish the severity of invasion. **F** Reduction in infiltrative extent following microtubule inhibitor treatment in in vivo models. Colchicine-treated group exhibited greater decrease in invasiveness than BKM120-treated group, however, this was not statistically significant (*p*-value=0.39, Fisher’s exact test). **G** Orthotopic xenografts treated by microtubule inhibitor (colchicine) exhibited improved survival outcome compared to vehicle-treated xenografts, while BKM120 treatment failed to show statistically significant improvement of prognosis. Survival analysis was performed using a Kaplan–Meier plot and the log-rank test was used to show statistical differences between survival curves. Col, colchicine; Vin, vinorelbine; n.s., non-significant.

Several genomic signatures associated with cytoskeletal microtubule assembly were identified by gene set enrichment analysis in GBM patients with *PTEN* edge mutations (Supplementary Fig. S11). This finding was further corroborated at the protein level, with several cytoskeletal proteins highly expressed in U87MG cells overexpressing PTEN edge mutants (Supplementary Table S1). Colchicine, a microtubule inhibitor, was used for further experiments. PDCs expressing edge mutants exhibited significantly fewer invasive cellular projections following colchicine treatment (Fig. 5A). Moreover, they underwent morphological changes in response to microtubule inhibitors in accordance with a decrease in invasive branches as shown in microfluid assay (Fig. 5B). BKM120 only disrupt the invasive phenotype of *PTEN*-null PDCs (*p*-value=0.044, one-sided *t* test). Neither BKM120 nor colchicine failed to disrupt the invasive capacity of PDCs with nuclear mutation (*p*-value=0.13 and 0.292 for BKM120 and colchicine, respectively, one-sided *t* test).

We observed that tubulin aggresomes were more frequent in U87MG cells with edge mutants. These aggresomes co-localized with PTEN at cellular margins, thus, implying the degradation of mutant proteins following microtubule inhibitor treatment (Fig. 5C). PDCs with endogenous edge mutations also showed the same changes following BKM120 and microtubule inhibitor treatment (Fig. 5D). These results imply that edge-mutant phenotypes are more related to cytoskeletal assembly than to PI3K/Akt signaling, suggesting microtubule inhibitors as a possible therapeutic option for GBMs with *PTEN* edge mutations.

To validate these findings in vivo, we established orthotopic xenograft models with a tumor bearing the *PTEN* edge mutation R130Q (P090-R130Q). A previous study demonstrated that prognosis of xenograft models could be less correlated to their in vitro invasive phenotype^37^. Therefore, we employed binary classification to determine the severity of invasion on histologic specimen. (Fig. 5E). Colchicine treatment showed a greater decrease in invasiveness than did BKM120 treatment. Colchicine-treated xenografts exhibited improved prognosis (*p*-value = 0.005, Log-rank test) (Fig. 5F, G).

### Clinical relevance of PTEN edge mutations

**Fig. 6:**
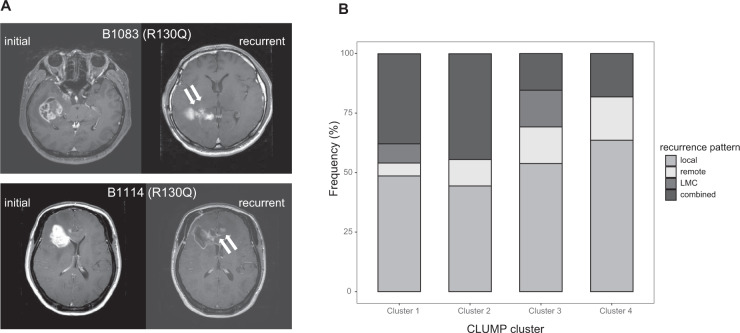
Radiographic failure patterns of GBM tumors with *PTEN* edge mutations. **A** Representative MR images depicting the locally invasive failure pattern of GBM tumors with *PTEN* edge mutation. Two GBM patients with *PTEN* edge mutation R130Q demonstrated locally invasive failure patterns, with tumors transmitted to the contralateral hemisphere in a locally invasive, infiltrative manner. **B** Distribution of radiographic recurrence patterns in CLUM-defined clusters. Edge mutations were included in cluster 4, while, the nuclear mutation was included in cluster 1 with other mutations in the C2 domain. LMC, leptomeningeal seeding.

To address clinical relevance, we retrospectively reviewed the patients’ records and radiographic data. Interestingly, patients with *PTEN* edge mutations had locally invasive radiographic phenotypes upon recurrence (Fig. 6A). However, the number of patients was limited by the small size of the total cohort, we adapted CLUMP-defined mutation clusters to measure the incidence of distinct failure patterns as a function of *PTEN* mutations.

We categorized the patients by CLUMP-defined clusters and compared the landscapes of distinct failure patterns. Local recurrence was the most predominant pattern in patients in cluster 4, which included the edge mutations, while combined recurrence was more prominent in patients in cluster 1, which contained the nuclear mutation, along with mutations in the C2 domain (Fig. 6B). Interestingly, mutations in the phosphatase domain could further subdivided into CLUMP clusters 2, 3, and 4, with 2 and 3 exhibiting different patterns of recurrence compared to cluster 4. GBM patients with *PTEN* mutations, regardless of the type or residue, had increased incidence of the combined type of recurrence pattern (30.1% with *PTEN* mutations vs. 18.3% in PTEN-WT, *P*-value = 0.071, Fisher’s exact test) (Supplementary Fig. S12). We assumed that this predominance was not attributed to edge mutations, but to other phosphatase domain mutations. In other words, the phenotypical relevance of *PTEN* mutations could vary by mutation residue, even within the phosphatase domain.

## Discussion

Our study found distinct phenotypes caused by different *PTEN* mutations. Analysis of the mutational landscape of GBM patients revealed that *PTEN* mutations could be categorized into two major subsets, missense mutations in the phosphatase domain and truncations of the C2 domain. The dimeric structure of PTEN has been shown to be critical for its catalytic function^2^, consequently, *PTEN* missense mutations exhibited dominant-negative effects over wild-type *PTEN*. In other words, a missense mutation is likely more disastrous than *PTEN* deletion or *PTEN*-destabilizing mutations in the context of malignancy. However, we failed to observe a statistically significant difference in survival of GBM patients with different *PTEN* alterations. As survival of GBM patients is confounded by multiple factors, we were prompted to evaluate the functional properties of *PTEN* mutations

PTEN function varies as a function of subcellular compartmentalization^21^; for example, nuclear PTEN is known to be associated with chromatin stability, as well as with DNA repair^18,19^. Unexpectedly, *PTEN* mutations evaluated in this study, despite their similar locations within the phosphatase domain and the same types of mutation, differed in their subcellular location, suggesting distinct functions (edge vs. nuclear). The crystal structure of the PTEN protein showed that all residues affected by edge mutations are located within the same pocket of the phosphatase domain, thus implying a similar effect on catalytic function. CLUMP analysis also distinguished edge mutations from nuclear mutations, in accordance with the above finding.

Interestingly, PTEN edge mutants were found at the cellular edges in response to chemotaxis, which was contrary to previous notions. PI3K and PTEN, which regulate the phosphoinositide signaling pathway, are spatially modulated to regulate the polarity of migrating cells. Studies using *Dictyostelium discoideum* demonstrated reciprocal spatial distribution of PI3K and PTEN at the leading edge and the rear of chemotaxing cells, respectively^22^. A study on human cells demonstrated a homogeneous distribution of PTEN in migrating cells^38^. Our findings were not consistent with these results, thus suggesting the peculiar function of PTEN edge mutants regarding the cellular locomotion.

We confirmed the invasive phenotypes of these edge mutations in vitro, including the development of cellular projections. To address the underlying mechanism of this phenotypical consequence, we applied the pan-PI3K/Akt inhibitor BKM120. Surprisingly, invasive phenotypes derived from edge mutations were not affected by the BKM120 treatment. Rather, they are inhibited by microtubule inhibitors, suggesting aberrant cytoskeletal assembly as a potent mechanism of action underlying the invasion attributed to edge mutations.

The role of PTEN in cellular migration and invasion is usually described with an emphasis on actin dynamics^28^. We observed that PTEN edge mutants were co-localized with F-actin at the leading edges of the motile cell. Regulation of phosphoinositides by PTEN is the major contributor to actin dynamics^28^, however, the invasive phenotype, reflected by increased cellular projections, was not disrupted by an anti-PI3K/Akt agent, suggesting the existence of another underlying mechanism.

Microtubules are one of the major components underlying cellular migration; however, their role in the perspective of PTEN function has not been well elucidated. Interactions between integrin and the extracellular matrix are important in several cellular processes, including motility, and focal adhesion usually mediates cellular migration with dynamic microtubules^39^. Focal adhesion kinase (FAK) is involved in this process and serves as one of the major substrates for PTEN^8^. Therefore, we hypothesized that microtubule assembly was likely a relevant biological process involved in *PTEN* mutation-derived cellular locomotion. A recent study on neuronal growth demonstrated that *PTEN* alteration status was associated with the detyrosinated status of microtubules, relating it to axon outgrowth^40^. We found that a microtubule inhibitor successfully abrogated the invasive phenotypes driven by *PTEN* edge mutations, largely consistent with their findings. However, the authors noted that neither PI3K nor FAK signaling fully accounted for the PTEN-regulated axonal growth; thus, the mechanism of action for microtubule assembly driving PTEN-associated cellular function, including migration and neuronal growth, should be further evaluated in future studies.

Recently, Chen et al. demonstrated the symbiotic relationship between macrophage and glioma cells in *PTEN*-null GBM^41^. According to their study, *PTEN*-null GBMs promote the recruitment of macrophage through the YAP1-LOX-β1 integrain-PYK2 axis, thus contributing the hostile tumor microenvironment (TME) reflected by poor prognosis. Enhanced tumor invasion attributed to *PTEN* edge mutations during chemotaxis suggests the possible role of *PTEN* mutations in the context of TME. Development of cellular projections as well as increased cellular locomotion during the chemotaxis of GBMs with PTEN edge-mutant might be indirect cues for their inter-connected roles with surrounding TME. As shown in Chen et al, *PTEN* deleted or mutated GBMs harbored more proportion of the immune cell population, which mainly composed of macrophage. This implies that immune cells might be the most relevant counterparts for the invasive *PTEN*-mutant GBM tumor cells. Future studies should focus on unraveling the role of *PTEN* mutations within the context of TME.

The present study illustrated the varied functional properties of *PTEN* missense mutations. Even substitutions in the same domain were further divided into subgroups with distinct subcellular localization. We found that edge mutants exhibited invasive phenotypes associated with dysfunctional cytoskeletal assembly, suggesting a microtubule inhibitor as a therapeutic option for these mutant-harboring GBMs. Additionally, we explored the clinical relevance of different *PTEN* mutations. A CLUMP-defined subgroup containing the edge mutations showed a locally invasive recurrence pattern, while patients with any *PTEN* mutation were mostly exhibited the combined failure pattern, which was totally different from local invasion. This finding further corroborates our hypothesis of mutation-specific functional characteristics of *PTEN* mutations and suggests that a differential therapeutic approach is necessary according to mutation type.

We evaluated the limited number of *PTEN* mutations for their functional characteristics in the current study and found that edge mutations exhibited peculiar subcellular localization and invasive property. Edge mutations exhibited enhanced invasion capacity compared to *PTEN*-deletion or non-edge mutation, however, the severity of invasion differed by mutation. Especially, H93Y mutation showed a variable degree of invasion, and the statistical significance of its superior invasiveness was only confirmed in in vitro 3D spheroid invasion assay (Fig. 3E). However, mice with H93Y mutation showed worse prognosis (Fig. 3C) and in vitro microtubule sensitivity assay supported that invasive phenotype of this mutation shared the same biological pathway with other edge mutations (Fig. 5A). Phenotypical penetrance of genotype can be various. We speculated that H93Y mutation might have a more variable degree of penetrance regarding both in vitro and in vivo perspectives. This finding further corroborates the mutation-specific functional implication, thereby signified the importance of a mutation-specific therapeutic approach to fulfill the unmet needs for precision oncology.

In summary, we observed that *PTEN* mutations exhibit distinct functional properties in accordance with their subcellular localization. Some missense mutations in the phosphatase domain, named edge mutations, caused enhanced invasiveness, which was associated with a dysfunctional cytoskeletal assembly. Clinically, mutation-specific therapeutic options should be considered in treating GBM patients with *PTEN* mutations.

## Materials and methods

### Glioblastoma specimens and their derivative cells

After obtaining informed consent, GBM tumor specimens and clinical records were obtained from patients undergoing surgery at the Samsung Medical Center. This study was approved by local Institutional Review Board (IRB) (IRB no. 2010-04-004, Samsung Medical Center, Seoul, Republic of Korea). All study process was conducted in accordance with the recommendations of the Declaration of Helsinki for biomedical research involving human subjects (1975). Surgical samples were snap-frozen using liquid nitrogen for genomic analysis. Tumor cells were cultured in neurobasal medium with N2 and B27 supplements (0.5X, each), human recombinant basic fibroblast growth factor (bFGF), and epidermal growth factor (EGF). The patient-derived cells used here had shown no obvious contamination of mycoplasma. The human glioma cell line U87MG was purchased from ATCC and cultured in MEM (GIBCO) containing 10% FBS (GIBCO).

### Data Generation from DNA and RNA sequencing

Using data from whole-exome and targeted-DNA sequencing, we detected single-nucleotide variants and indels and estimated copy number in GBM samples. RNA sequencing data were used to determine gene expression and gene fusion events. PTEN mutation profiles are presented in Supplementary Table S2 and samples used for transcriptomic analysis have been deposited in the European Genome-phenome Archive (EGA) under accession EGAS00001004753. *PTEN* mutation of TCGA samples was from provisional TCGA data which were downloaded from cBioportal (www.cbioportal.org).

#### Whole-exome and target DNA sequencing

Genomic DNA was extracted from fresh tissue specimens using the QIAamp DNA kit (Qiagen, Valencia, CA, USA). The sequenced reads in the FASTQ files were aligned to the human genome assembly hg19 using the Burrows-Wheeler aligner version 0.6.2 (BWA). The initial alignment BAM files were pre-processed for sorting, removing duplicate reads, realigning reads around potential small indels, and recalibrating base quality scores using SAMtools. MuTect and Somatic IndelDetector (GATK version 2.2) were used to make high-confidence predictions on somatic mutations from neoplastic and non-neoplastic tissue pairs. The variant effector predictor (VEP) was used to annotate somatic mutations.

#### RNA sequencing, differential gene expression analysis (DEG), and gene set enrichment analysis (GSEA)

For all samples, RNA-seq libraries were prepared using the Illumina TruSeq RNA Sample Prep kit. For analysis of mRNA expression levels, sequenced reads in FASTQ files were trimmed to include only 30 nucleotides from the 5′ end of each read. The trimmed reads were mapped on hg19 using GSNAP, version 2012-12-20^38^. The resulting alignment SAM files were sorted and summarized into BED files using SAMtools and bedTools (bamToBed version 2.16.2)^39^. The BED files were used to calculate reads per kilobase of transcript per million reads (RPKM) of mapped values for each gene by using the R package DEGseq^40^ and the RefSeq gene annotations (refFlat table, downloaded from the University of California, Santa Cruz [UCSC] Genome Browser, last accessed on August 6, 2012).

To explore the transcriptomic signatures associated with *PTEN* edge mutations, gene set enrichment analysis was performed using R packages. Genes which were differentially expressed in GBM tumors with *PTEN* edge mutations were identified (*‘DEGseq’*) and used to identify the GO gene sets significantly enriched in these samples(“*clusterProfile*”).

### Xenograft mouse model

All in vivo experiments were conducted in accordance with the guideline of the Association for Assessment and Accreditation of Laboratory Animal Care and approved by Samsung Medical Center Animal Use and Care Committee (IRB file no. 20160112002). We propagated GBM PDXs to evaluate the effect of different PTEN mutants by implanting U87MG cell lines with distinct *PTEN* mutations (*PTEN-*null, C124S, H93Y, R130Q, and R173C) into the flanks of 6–8 weeks female BALB/c-nude mice, which were purchased from Orient Bio Inc (Seongnam, Korea). Three *PTEN*-null xenografts were established and six mice per each *PTEN* mutation were established. Survival was analyzed by Kaplan–Meier curves. *P*-values were calculated by the log-rank test.

To establish orthotopic xenograft models, 6–8 weeks female BALB/c-nude mice (Orient Bio Inc.) were used for intracranial implantation. Patient-derived GBM tumor cells with R130Q *PTEN* mutation (P090-R130Q) were dissociated and suspended with Hank’s Balanced Salt solution (HBSS; 14170-122, Gibco). A total of 2 ×10^5^ cells (5uL) were injected intracranially to each mouse using a rodent stereotactic frame. Orthotopic PDXs were stratified by body weight and then randomly assigned to distinct treatment groups; 6 mice per each treatment group respectively. BKM120 and colchicine were resuspended in NMP (N-methyl-2-pyrrolidone): PEG300 1:9 v:v and PBS, respectively^33,41,42^. BKM120 was administered by oral gavage at 20 mg/kg and 40 mg/kg on 5 days on, 2 days off schedule^33,41^. Colchicine was administered by intraperitoneal injection of 0.5 mg/kg once a week^42^. All treatments were administered until euthanasia. Animals showing moribund status or loss of body weight > 20% were sacrificed. Whole brains were extracted and formalin-fixed to make paraffin embedded tissue (FFPE) block for histologic analysis. Survival analysis and immunohistochemical studies were performed under blinded inspection.

### Establishment of PTEN mutant cell lines

The *PTEN* wild-type and C124 constructs were purchased from Addgene. To generate *PTEN* mutant constructs, the *PTEN* wild-type coding region was amplified using mutation-specific primers. The primers are specified in Supplementary Table S3. The amplified products were digested with DpnI (NEB) and ligated with T4 DNA ligase (NEB). To transfer the PTEN coding regions into pLenti6/V5-DEST (Invitrogen) and pCW57.1 (Addgene) plasmids, gateway LR reactions were carried out according to the manufacturer’s manual (Invitrogen). Sequences of these constructs were confirmed by Sanger sequencing. PTEN constructs were co-transfected with lentiviral packaging plasmids (VSVG and PAX2, Addgene) and then, *PTEN-*null GBM cells were infected by these lentiviral vectors. We sorted the infected GBM cells by selectively killing uninfected cells by puromycin or blasticidin (InvivoGen).

### Invasion assay

We performed three in vitro invasion assays, trans-well invasion assay, 3D spheroid invasion assay and microfluidic invasion assay. For trans-well invasion assay, U87MG cells in serum-free medium were seeded into the Matrigel Invasion chamber with 8.0 um PET Membrane (Corning, Inc.). Cells which invaded the lower surface of the insert were fixed with methanol and then stained with Harris hematoxylin and eosin. For 3D spheroid invasion assay, an invasive matrix (Trevigen, Inc.) was added into each well where a neurosphere was embedded and then, both chemoattractant medium and inhibitors were added. Invasive branches from spheroids were evaluated after 3–6 days. A microfluidic assay was carried out in accordance with the previous literature^42^.

### Western blotting

GBM cells were harvested and lysed using NP-40 lysis buffer (Invitrogen), which consisted of 1% NP-40, 5% glycerol, 20 nmol/L NaF, 5 mmol/L EDTA, 5 mmol/L EGTA, freshly added HaltTM protease and phosphatase inhibitor cocktail (Thermo Scientific), and 1 mM PMSF (Sigma). The membrane was incubated with primary antibodies against the protein of interest at 4 °C overnight. The signals from the antibodies were amplified by HRP-conjugated secondary antibodies and detected with ECL solution (Thermo Scientific). The following antibodies were used: PTEN (Cell signaling), phospho-Akt (Cell signaling), Akt (Cell signaling), phospho-FAK (Cell signaling), FAK (Cell signaling), and GAPDH (Abcam).

### Immunofluorescence

Chamber slides (Lab-tek) were pre-coated with poly-L-ornithine (Sigma), and single cells were seeded 4 h before the experiments. To stain cells, they were fixed with 4% paraformaldehyde, and blocking solution (5% NHS and 0.5% Triton X-100 in PBS) was added at room temperature for 1 h. Then, the cells were incubated with antibodies diluted in 1% BSA and 0.3% Triton X-100 in PBS. The following antibodies were used as primary antibodies: PTEN (Cell signaling), V5 (Abcam), CytoPainter Phalloidin-eFluor 647 (Abcam), tubulin (Abcam), and Ki67 conjugated to Alexa Fluor 647 (BD Pharmingen). Analysis was performed using confocal LSM 700 and LSM780 microscopy (Carl Zeiss).

### Transmission electron microscopy (TEM)

GBM cells were plated in laminin-coated 6-well plates and incubated at 37 °C. When the cells reached 70-90% confluence, a fixative (2% paraformaldehyde and 2.5% glutaraldehyde in 0.1 M phosphate buffer) was added and incubated overnight at 4 °C. Fixed cells were washed with 0.1 M phosphate buffer several times and then post-fixed in 1% osmium tetroxide for 1 hr. *En bloc* staining was carried out using 0.1% uranyl acetate in 50% ethanol for 1 h and dehydrated in an ethanol series, followed by embedding in Epon 812 resin (Okenshoji Co., Ltd). Sections were cut to 70 nm using an ultra-microtome (EM-UC7, Leica) and stained with 2% uranyl acetate for 20 min, followed by Reynold’s lead citrate for 5 min. The samples were observed under a Hitachi H-7500 TEM at 80 kV acceleration voltage.

### Mass spectrometry

PTEN mutant-overexpressing U87MG cell lines were lysed in NP40 lysis buffer. Then, immunoprecipitation was conducted using anti-PTEN antibody (Cell Signaling Technology). The immunoprecipitated lysate was subjected to proteolysis and LC-MS/MS. The tryptic peptides were analyzed using a LTQ‐XL mass spectrometer (Thermo Scientific, San Jose, CA, USA), coupled with the Agilent 1200 HPLC system (Agilent technology) at a flow rate of 0.4 μL/min, with a linear gradient of acetonitrile from 5 to 40% in water in the presence of 0.1% formic acid for 40 min. The MS survey was scanned from 300 to 2000 m/z, followed by three data-dependent MS/MS scans with the following options: isolation width, 1.5 m/z; normalized collision energy, 25%; dynamic exclusion duration, 180 s. The acquired MS/MS spectra were searched against the UniProt human database (April 2014 release) using SEQUEST in Proteome Discoverer 1.4 (Thermo Fisher Scientific, Waltham, MA, USA, version 1.4.0.288). Search parameters were two missed trypsin cleavage sites, cysteine carbamidomethylation (+57.0215 Da) as fixed modifications, and methionine oxidation (+15.9949 Da) as a variable modification. Peptide and protein results were filtered to less than a false discovery rate (FDR) 1%.

### Invasion scoring system

We adopted the criteria to assess the degree of invasion from the literature^37^. Originally, authors defined the degree of invasion on the basis of hematoxylin-eosin (H&E) stained sections as follows:

**Table:** 

Invasion score	Description
0	mild diffuse invasion, rather sharply delineated tumor bulk
1	mild diffuse invasion, rather sharply delineated tumor bulk with mild invasion of the corpus callosum
2	moderate to strong diffuse infiltration of one brain hemisphere
3	massive diffuse infiltration of both hemispheres, gliomatosis-like pattern

It is hard to exactly quantify the extent of invasion between “moderate” and “strong” grade in our xenograft models; both grades exhibited the evident transmission to the contralateral hemisphere via corpus callosum, however, diffuse infiltration within the engraft hemisphere was more severe in “strong” grade. To make a clear distinction between histologic invasiveness categories, we applied binary classification, “non-invasive + mild” versus “moderate + strong”.

### Statistics

All statistical analyses were performed using R version 3.6.3 (http://www.R-project.org). Data are shown as the means of at least triplicates of experiments ± standard deviation (s.d). The exact number of experimental replicates is indicated in each figure legend. Continuous variables were compared using the two-sided independent sample *t* test. T test was performed after confirming the normality assumption using Shapiro-Wilk’s test. A two-sided Wilcoxon test was performed when appropriate for the non-parametric statistical test. And categorical variables were tested using Fisher’s exact test. Survival analysis was performed using a Kaplan–Meier plot, and the log-rank test was used to show statistical differences between survival curves. Statistical methods applied for each figure were indicated in figure legends.

*P*-value ≤ 0.05 was used as a threshold for statistical significance. *P*-values derived from multiple comparisons were appropriately corrected by false discovery rate (FDR) methods. *P*-values were rounded off to three decimal places. *P*-values were presented in following format; n.s, *P*-value > 0.05; *, *P*-value ≤ 0.05; **, *P*-value ≤ 0.01; and ***, *P*-value ≤ 0.001.

## Supplementary information

Supplementary Figure Legends

Supplemenatry Figure S1

Supplemenatry Figure S2

Supplemenatry Figure S3

Supplemenatry Figure S4

Supplemenatry Figure S5

Supplemenatry Figure S6

Supplemenatry Figure S7

Supplemenatry Figure S8

Supplementary Figure S9

Supplementary Figure S10

Supplementary Figure S11

Supplementary Figure S12

Supplementary table S1

Supplementary table S2

Supplementary table S3

## Data Availability

The datasets used for transcriptomic analysis have been deposited in the European Genome-phenome Archive (EGA) under accession EGAS00001004753. Other data analyzed during the current study are available from the corresponding author on reasonable request.
