# Supplementary material for: Mutation-specific non-canonical pathway of PTEN as a distinct therapeutic target for glioblastoma
Source: Cell Death Dis. 2021 Apr 7;12(4):374. doi: 10.1038/s41419-021-03657-0 (PMC8027895; doi:10.1038/s41419-021-03657-0)
Supplement: Supplementary file 1 — Supplementary Figure Legends [file 41419_2021_3657_MOESM1_ESM.docx]

**Supplementary figure legends**

**Supplementary Figure S1.** Mutational landscape of the glioblastoma(GBM) patients in this study cohort (Oncoprint)

**Supplementary Figure S2.** Mutation profiles of *PTEN* in GBM tumors (TCGA, provisional) Statistically significant association between mutation type and *PTEN* domain was also validated in TCGA data set (p-value <0.001, Fisher’s exact test).

**Supplementary Figure S3.** Survival outcome of GBM patients in TCGA cohort stratified by *PTEN* alteration status.

*Abbreviation*: OS, overall survival; mos, months; WT, wild-type; DEL only, deletion only; MUT only, mutation only; both, both deletion and mutation alleles.

**Supplementary Figure S4**. P-Akt activity of *PTEN*-null PDCs (P090) overexpressing wild-type and mutant PTEN.

**Supplementary Figure S5.** Subcellular compartmentalization of PTEN mutants according to distinct CLUMP-defined clusters. CLUMP analysis of *PTEN* mutations identified several functional subgroups and CLUMP cluster 4 and cluster 1 were respectively highlighted by edge mutation and nuclear mutation. Some of PTEN mutants of phosphatase domain, which were included in the different cluster, exhibited the same subcellular localization in accordance with CLUMP-defined clusters, further corroborating the functional relevance of CLUMP clusters. Cellular periphery localization of PTEN G129E mutant (CLUMP cluster 4) (central); nuclear localization of PTEN Y177C mutant (CLUMP cluster 1) (left).

**Supplementary Figure S6**. Survival analysis of orthotopic xenograft models established by U87MG cells with *PTEN*-null and nuclear mutation (R173C). Survival analysis was performed using a Kaplan–Meier plot, and the log-rank test was used to show statistical differences between survival curves.

**Supplementary Figure S7.** *In vitro* trans-well invasion assay to evaluate the invasion capacity of U98MG cells with various *PTEN* mutations.

**Supplementary Figure S8.** *In vitro* microfluidic assay to evaluate the invasion capacity of *PTEN*-null PDCs (P089) cells with various *PTEN* mutations.

**Supplementary Figure S9.** Co-localization of PTEN edge mutants and F-actin at the leading edge of motile cells (*PTEN*-null PDCs with exogenous edge mutants) upon chemotactic stimuli.

**Supplementary Figure S10.** Co-localization of PTEN edge mutants and F-actin at the leading edge of motile cells (PDCs with endogenous edge mutants) upon chemotactic stimuli.

**Supplementary Figure S11.** Gene set enrichment analysis(GSEA) of GBM tumors with *PTEN* edge mutations. Screened genes, which significantly upregulated in GBM tumors with edge mutations compared to those with other types of *PTEN* mutations were used for GSEA. Several gene sets associated with cytoskeletal assembly, including intermediate filaments and microtubules, were identified as enriched genomic signatures in GBM tumors with edge mutations.

*Abbreviation*: FDR, false discovery rate

**Supplementary Figure S12**. Distribution of radiographic recurrence patterns in GBM patients stratified by *PTEN* status. Right, recurrence pattern in PTEN-mutant GBM patients; Left, recurrence pattern in PTEN-wild-type GBM patients.

*Abbreviation*: LMC, leptomeningeal see
