# Supplementary figures and images for: Mutation-specific non-canonical pathway of PTEN as a distinct therapeutic target for glioblastoma
Source: Cell Death Dis. 2021 Apr 7;12(4):374. doi: 10.1038/s41419-021-03657-0 (PMC8027895; doi:10.1038/s41419-021-03657-0)

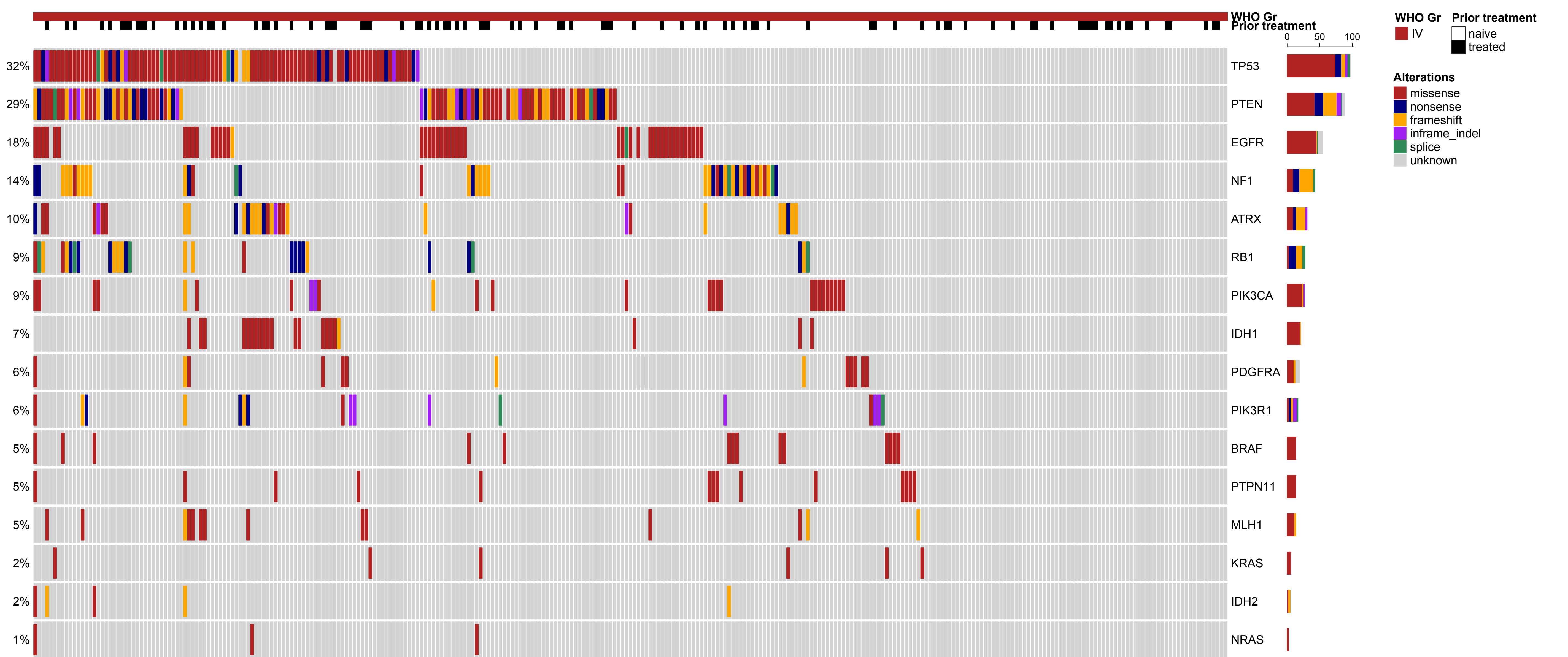

Supplement: Supplementary file 2 — Supplemenatry Figure S1 [file 41419_2021_3657_MOESM2_ESM.pdf]

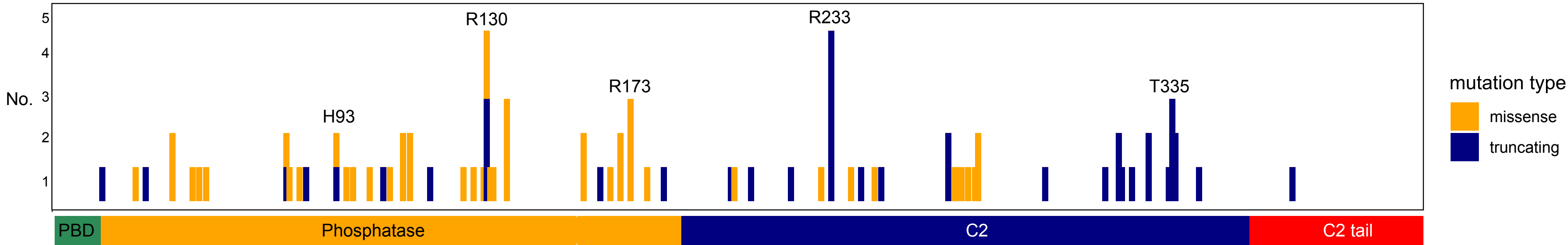

Supplement: Supplementary file 3 — Supplemenatry Figure S2 [file 41419_2021_3657_MOESM3_ESM.pdf]

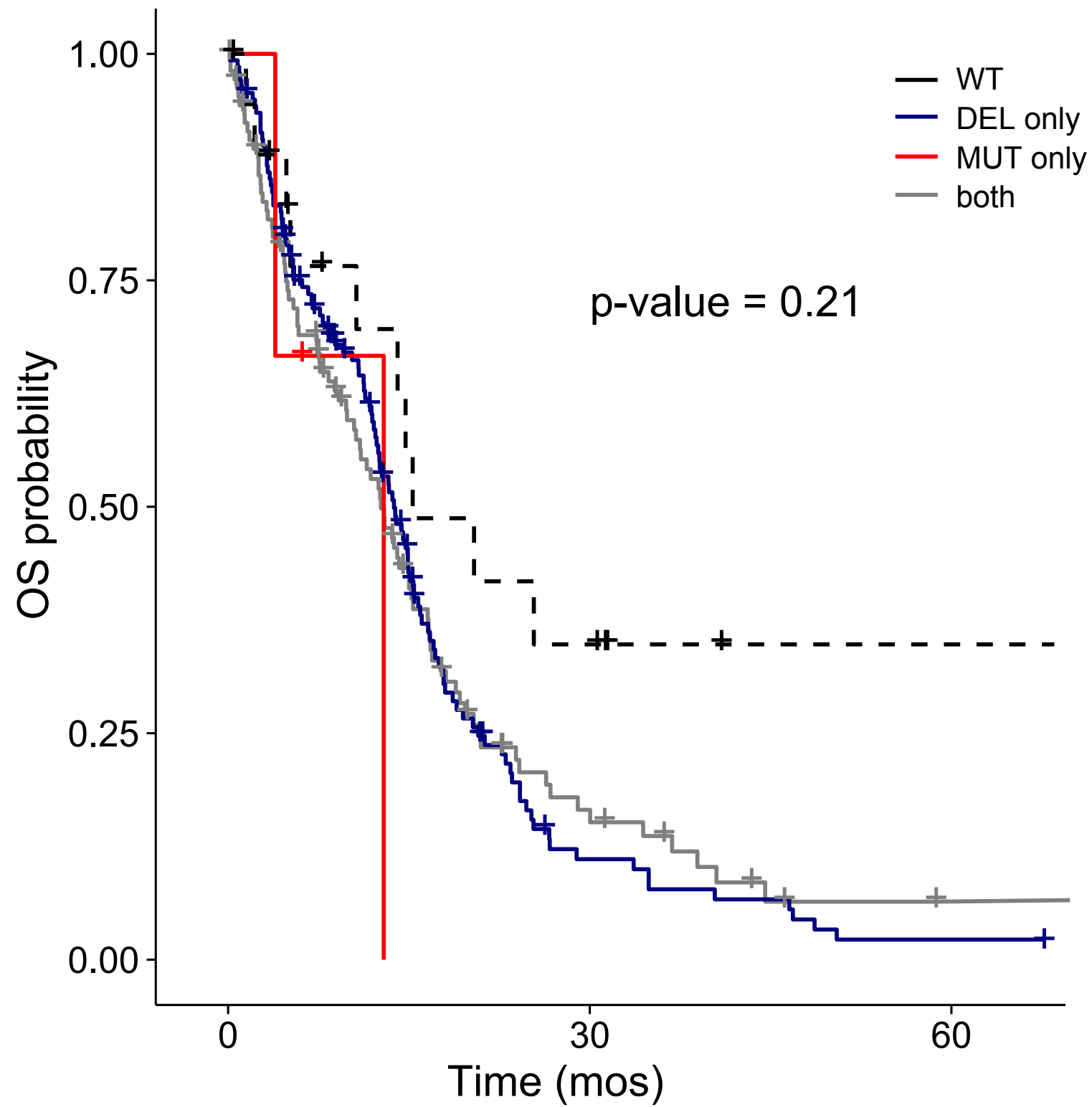

Supplement: Supplementary file 4 — Supplemenatry Figure S3 [file 41419_2021_3657_MOESM4_ESM.pdf]

## P090 (PTEN-null)

|         |    |    |      |       |       |
|---------|----|----|------|-------|-------|
| NT      | +  | -- | --   | --    | --    |
| PTEN-V5 | -- | +  | +    | +     | +     |
| Alter.  |    | WT | H93Y | C124S | R130Q |

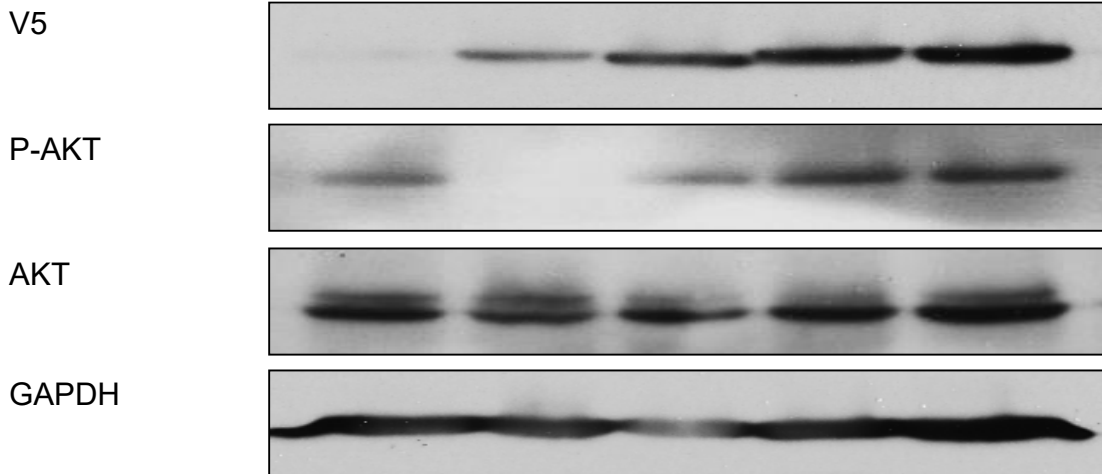

Supplement: Supplementary file 5 — Supplemenatry Figure S4 [file 41419_2021_3657_MOESM5_ESM.pdf]

U87MG-NT

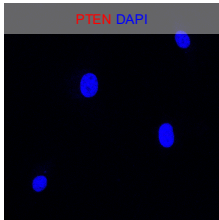

U87MG-G129E

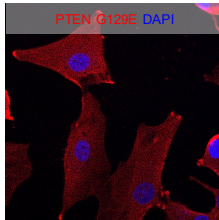

U87MG-Y177C

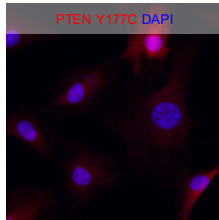

Supplement: Supplementary file 6 — Supplemenatry Figure S5 [file 41419_2021_3657_MOESM6_ESM.pdf]

Survival probability (OS)

U87-NT  
U87-R173C

P-value=0.033  
(log-rank test)

0

10

20

30

Time (days)

1.00  
0.75  
0.50  
0.25  
0.00

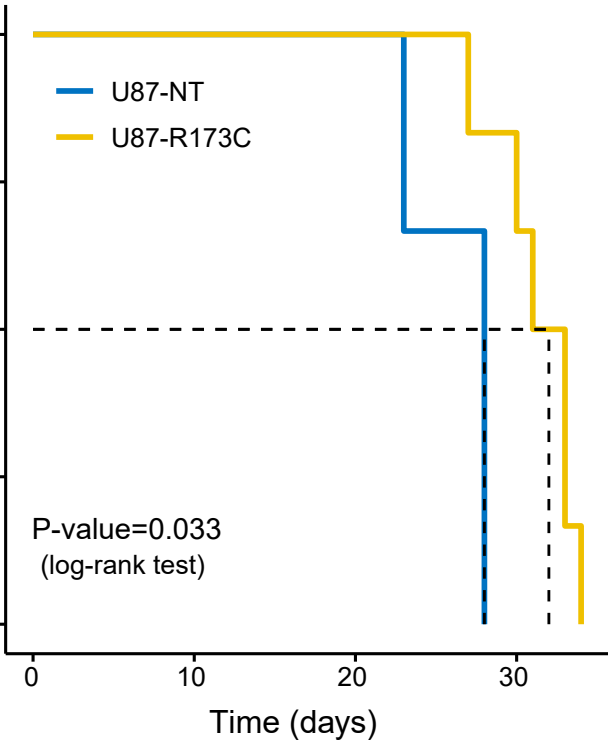

Supplement: Supplementary file 7 — Supplemenatry Figure S6 [file 41419_2021_3657_MOESM7_ESM.pdf]

# U87MG

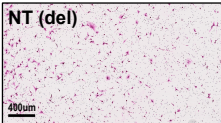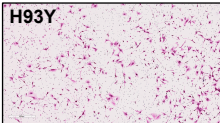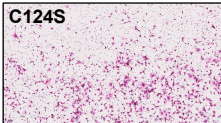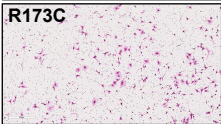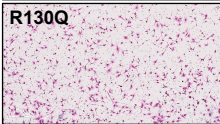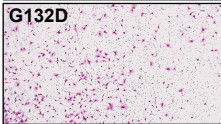

Supplement: Supplementary file 8 — Supplemenatry Figure S7 [file 41419_2021_3657_MOESM8_ESM.pdf]

# P089 (PTEN-null)

NT (del)

H93Y

C124S

R130Q

Day 0

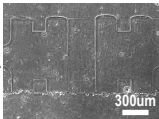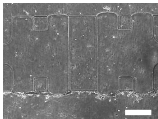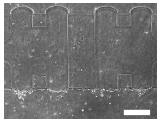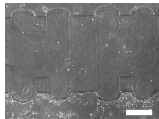

Day 4

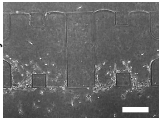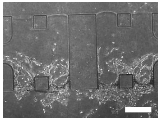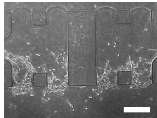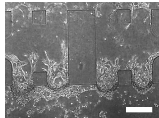

Supplement: Supplementary file 9 — Supplemenatry Figure S8 [file 41419_2021_3657_MOESM9_ESM.pdf]

P087 (endogenous H93Y)

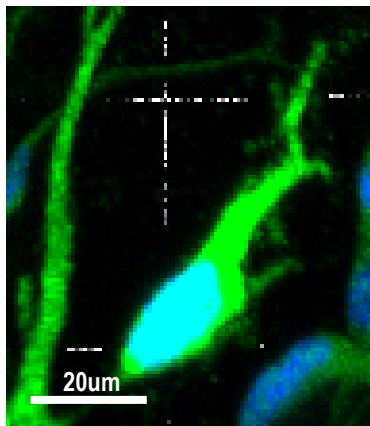

F-actin DAPI

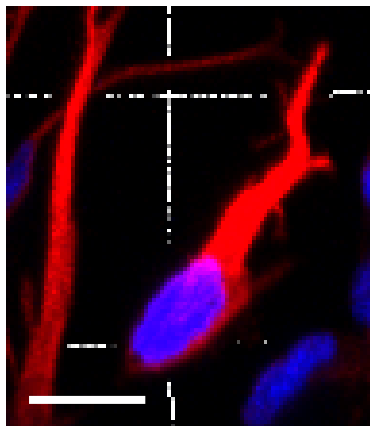

P045 (endogenous R130Q)

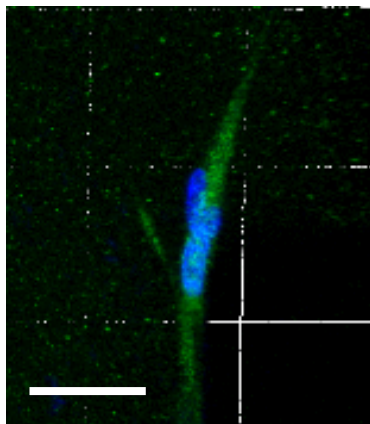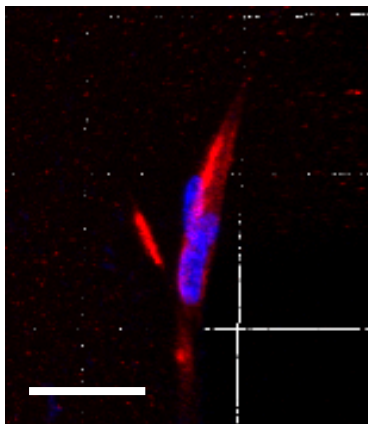

Supplement: Supplementary file 10 — Supplementary Figure S9 [file 41419_2021_3657_MOESM10_ESM.pdf]

**PTEN** **DAPI**

**PTEN** **F-actin**

**H93Y**

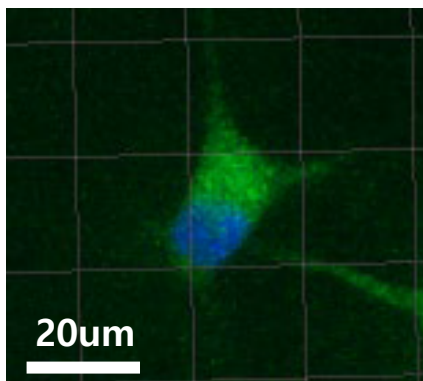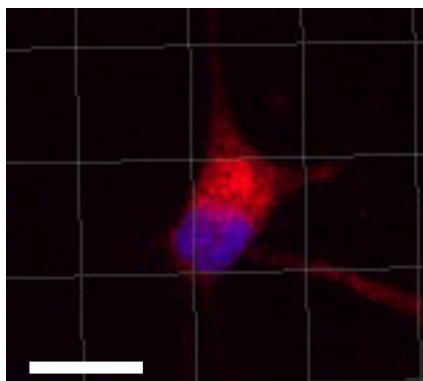

**C124S**

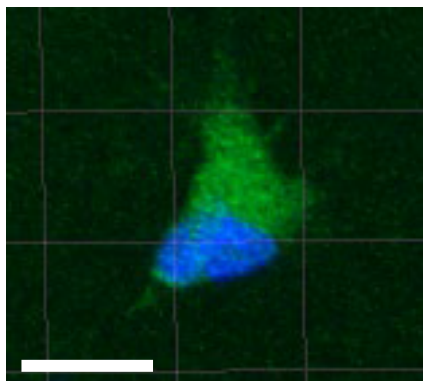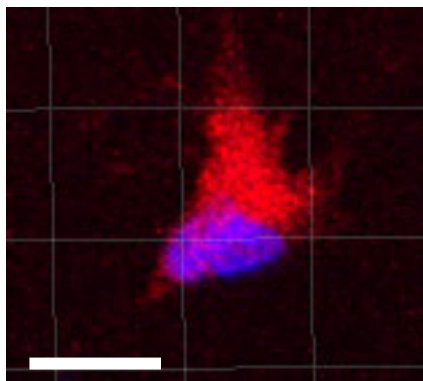

**R130Q**

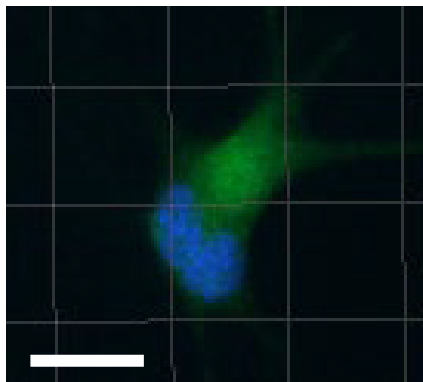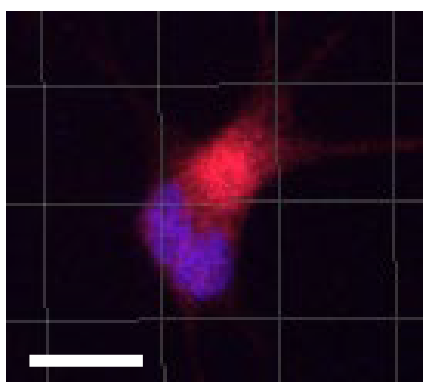

Supplement: Supplementary file 11 — Supplementary Figure S10 [file 41419_2021_3657_MOESM11_ESM.pdf]

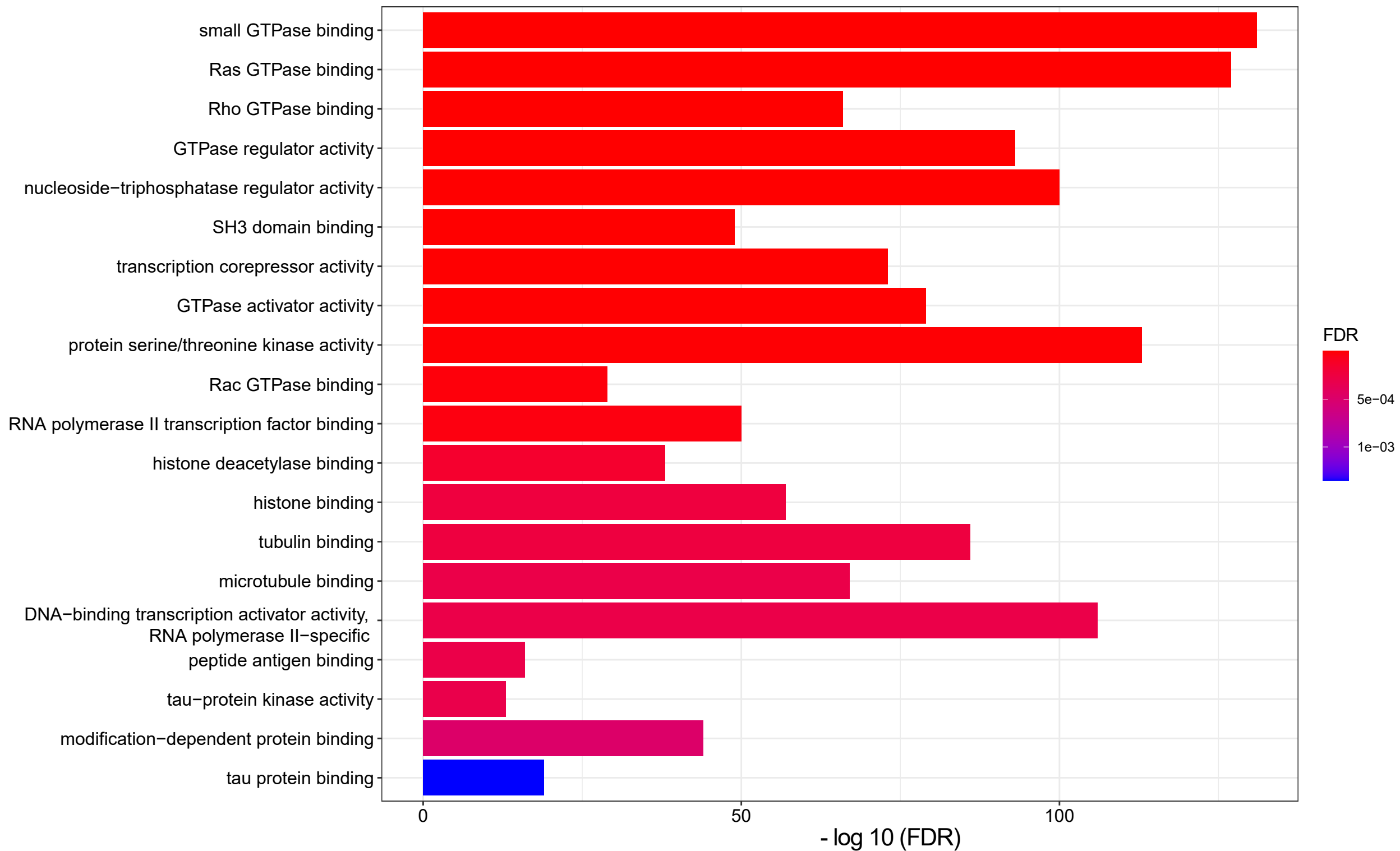

Supplement: Supplementary file 12 — Supplementary Figure S11 [file 41419_2021_3657_MOESM12_ESM.pdf]

mutant

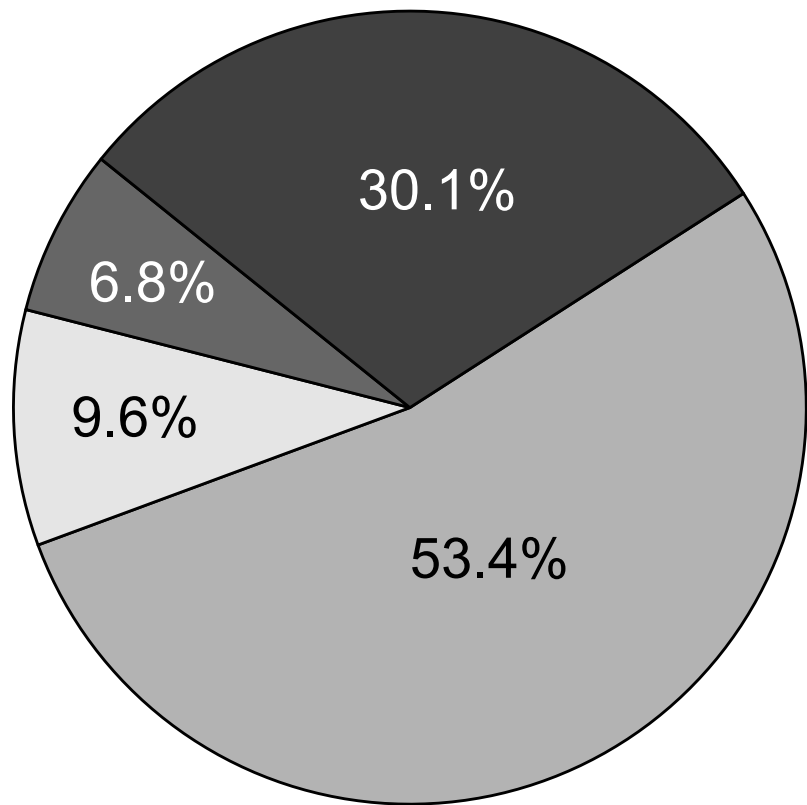

WT

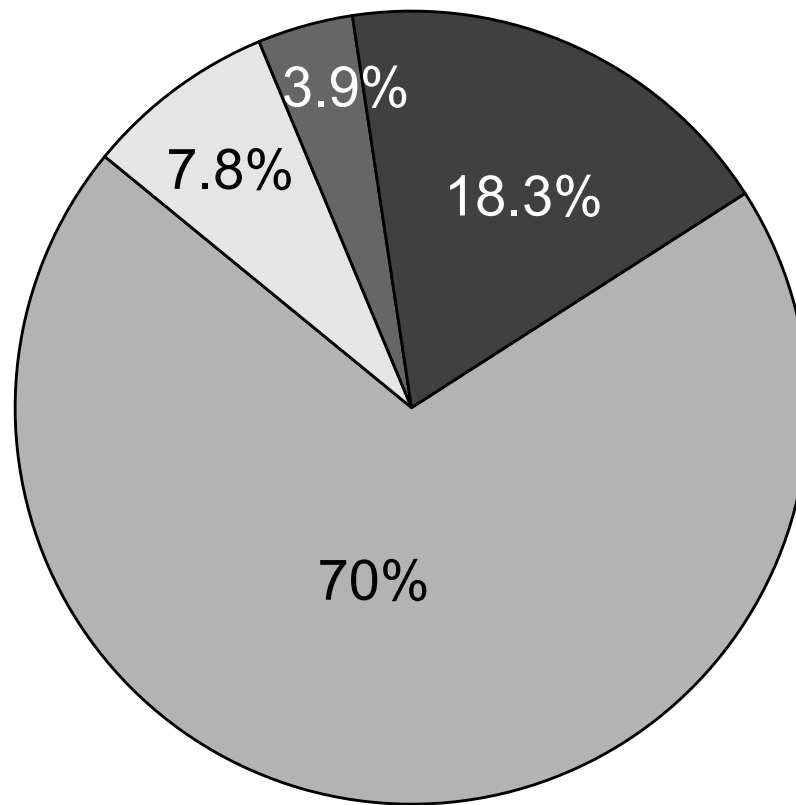

recurrence pattern

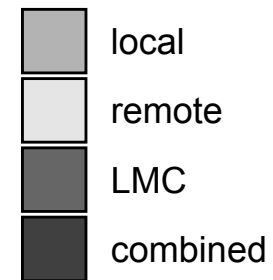

Supplement: Supplementary file 13 — Supplementary Figure S12 [file 41419_2021_3657_MOESM13_ESM.pdf]
