# Supplementary material for: Mutation-specific non-canonical pathway of PTEN as a distinct therapeutic target for glioblastoma
Source: Cell Death Dis. 2021 Apr 7;12(4):374. doi: 10.1038/s41419-021-03657-0 (PMC8027895; doi:10.1038/s41419-021-03657-0)
Supplement: Supplementary file 14 — Supplementary table S1 [file 41419_2021_3657_MOESM14_ESM.pdf]

Accession	Description	# Peptides				# PSM				Coverage				Score				GO		
		WT	H93Y	C124S	R130Q	WT	H93Y	C124S	R130Q	WT	H93Y	C124S	R130Q	WT	H93Y	C124S	R130Q	Gene ontology (biological process)	Gene ontology (molecular function)	Gene ontology (cellular component)
P08670	Vimentin OS=Homo sapiens GN=VIM PE=1 SV=4 - [VIME_HUMAN]	22	28	26	28	171	276	243	367	48.07%	60.52%	52.79%	57.51%	445.96	686.93	592.59	933.78	apoptotic process [GO:0006915]; astrocyte development [GO:0014002]; Bergmann glial cell differentiation [GO:0060020]; cellular component disassembly involved in execution phase of apoptosis [GO:0006921]; intermediate filament organization [GO:0045109]; lens fiber cell development [GO:0070307]; movement of cell or subcellular component [GO:0006928]; muscle filament sliding [GO:0030049]; negative regulation of neuron projection development [GO:0010977]; positive regulation of gene expression [GO:0010628]; SMAD protein signal transduction [GO:0060395]; viral process [GO:0016032]	double-stranded RNA binding [GO:0003725]; glycoprotein binding [GO:0001948]; identical protein binding [GO:0042802]; keratin filament binding [GO:1990254]; protein C-terminus binding [GO:0008022]; scaffold protein binding [GO:0097110]; structural constituent of cytoskeleton [GO:0005200]; structural constituent of eye lens [GO:0005212]	cell leading edge [GO:0031252]; cytoplasm [GO:0005737]; cytoskeleton [GO:0005856]; cytosol [GO:0005829]; extracellular vesicular exosome [GO:0070062]; focal adhesion [GO:0005925]; intermediate filament [GO:0005882]; intermediate filament cytoskeleton [GO:0045111]; neuron projection [GO:0043005]; peroxisome [GO:0005777]; plasma membrane [GO:0005886]
F6KCZ3	Phosphatase and tensin-like protein OS=Homo sapiens PE=4 SV=1 - [F6KCZ3_HUMAN]	7	7	8	7	15	55	30	38	24.81%	24.81%	27.05%	24.81%	39.24	141.85	75.18	101.76	inositol phosphate dephosphorylation [GO:0046855]; negative regulation of cell migration [GO:0030336]; negative regulation of cell proliferation [GO:0008285]; negative regulation of focal adhesion assembly [GO:0051895]; negative regulation of protein kinase B signaling [GO:0051898]; phosphatidylinositol-3,4,5-trisphosphate 3-phosphatase activity [GO:0016314]; phosphatidylinositol-3,4-bisphosphate 3-phosphatase activity [GO:0051800]; phosphatidylinositol dephosphorylation [GO:0046856]; protein tyrosine/serine/threonine phosphatase activity [GO:0008138]; protein tyrosine phosphatase activity [GO:0004725]; regulation of protein stability [GO:0031647]	magnesium ion binding [GO:0000287]; phosphatidylinositol-3,4,5-trisphosphate 3-phosphatase activity [GO:0016314]; phosphatidylinositol-3,4-bisphosphate 3-phosphatase activity [GO:0051800]; protein tyrosine/serine/threonine phosphatase activity [GO:0008138]; protein tyrosine phosphatase activity [GO:0004725]	
F6KCZ5	Phosphatase and tensin-like protein OS=Homo sapiens PE=4 SV=1 - [F6KCZ5_HUMAN]	6	7	8	6	14	49	27	29	20.10%	24.81%	27.05%	20.10%	36.54	121.95	67.02	74.17	inositol phosphate dephosphorylation [GO:0046855]; negative regulation of cell migration [GO:0030336]; negative regulation of cell proliferation [GO:0008285]; negative regulation of focal adhesion assembly [GO:0051895]; negative regulation of protein kinase B signaling [GO:0051898]; phosphatidylinositol-3,4,5-trisphosphate 3-phosphatase activity [GO:0016314]; phosphatidylinositol-3,4-bisphosphate 3-phosphatase activity [GO:0051800]; phosphatidylinositol dephosphorylation [GO:0046856]; protein tyrosine/serine/threonine phosphatase activity [GO:0008138]; protein tyrosine phosphatase activity [GO:0004725]; regulation of protein stability [GO:0031647]	magnesium ion binding [GO:0000287]; phosphatidylinositol-3,4,5-trisphosphate 3-phosphatase activity [GO:0016314]; phosphatidylinositol-3,4-bisphosphate 3-phosphatase activity [GO:0051800]; protein tyrosine/serine/threonine phosphatase activity [GO:0008138]; protein tyrosine phosphatase activity [GO:0004725]	
P35579	Myosin-9 OS=Homo sapiens GN=MYH9 PE=1 SV=4 - [MYH9_HUMAN]	5	1	3	6	6	1	5	10	4.85%	0.87%	2.60%	5.00%	16.51	2.67	14.35	28.21	actin cytoskeleton reorganization [GO:0031532]; actin-dependent ATPase activity [GO:0030898]; actin filament-based movement [GO:0030048]; actomyosin structure organization [GO:0031032]; angiogenesis [GO:0001525]; ATPase activity [GO:0016887]; axon guidance [GO:0007411]; blood vessel endothelial cell migration [GO:0043534]; cytokinesis [GO:0000910]; ephrin receptor signaling pathway [GO:0048013]; establishment of meiotic spindle localization [GO:0051295]; establishment of T cell polarity [GO:0001768]; integrin-mediated signaling pathway [GO:0007229]; in utero embryonic development [GO:0001701]; leukocyte migration [GO:0050900]; meiotic spindle organization [GO:0000212]; membrane protein ectodomain proteolysis [GO:0006509]; microfilament motor activity [GO:0000146]; monocyte differentiation [GO:0030224]; motor activity [GO:0003774]; myoblast fusion [GO:0007520]; negative regulation of actin filament severing [GO:1903919]; phagocytosis, engulfment [GO:0006911]; platelet aggregation [GO:0070527]; platelet formation [GO:0030220]; positive regulation of protein processing in phagocytic vesicle [GO:1903923]; protein transport [GO:0015031]; regulation of cell shape [GO:0008360]; termination of G-protein coupled receptor signaling pathway [GO:0038032]; uropod organization [GO:0032796]	actin binding [GO:0003779]; actin-dependent ATPase activity [GO:0030898]; actin filament binding [GO:0051015]; ADP binding [GO:0043531]; ATPase activity [GO:0016887]; ATP binding [GO:0005524]; microfilament motor activity [GO:0000146]; motor activity [GO:0003774]; poly(A) RNA binding [GO:0044822]; protein anchor [GO:0043495]; protein domain specific binding [GO:0019904]; protein homodimerization activity [GO:0042803]	actin cytoskeleton [GO:0015629]; actomyosin [GO:0042641]; actomyosin contractile ring [GO:0005826]; cell-cell adherens junction [GO:0005913]; cell leading edge [GO:0031252]; cleavage furrow [GO:0032154]; cytoplasm [GO:0005737]; cytosol [GO:0005829]; extracellular vesicular exosome [GO:0070062]; immunological synapse [GO:0001772]; membrane [GO:0016020]; myosin II complex [GO:0016460]; myosin II filament [GO:0097513]; neuromuscular junction [GO:0031594]; nucleus [GO:0005634]; plasma membrane [GO:0005886]; protein complex [GO:0043234]; ruffle [GO:0001726]; spindle [GO:0005819]; stress fiber [GO:0001725]; uropod [GO:0001931]
Q8N532	TUBA1C protein OS=Homo sapiens GN=TUBA1C PE=2 SV=1 - [Q8N532_HUMAN]	4	3	3	6	5	3	4	9	17.54%	13.85%	13.85%	24.92%	13.46	8.82	10.13	22.34	GTPase activity [GO:0003924]; microtubule-based process [GO:0007017]; protein polymerization [GO:0051258]	GTPase activity [GO:0003924]; GTP binding [GO:0005525]; structural constituent of cytoskeleton [GO:0005200]	cytoplasm [GO:0005737]; microtubule [GO:0005874]
E9PH82	Protein FAM98A OS=Homo sapiens GN=FAM98A PE=2 SV=1 - [E9PH82_HUMAN]	2	3	1	2	2	4	2	3	12.50%	16.99%	4.81%	9.29%	5.12	9.32	4.81	6.7			
O43175	D-3-phosphoglycerate dehydrogenase OS=Homo sapiens GN=PHGDH PE=1 SV=4 - [SERA_HUMAN]	1	1	3	2	1	1	3	2	3.00%	2.06%	6.57%	4.50%	2.37	2.21	6.76	4.83	brain development [GO:0007420]; cellular amino acid biosynthetic process [GO:0008652]; cellular nitrogen compound metabolic process [GO:0034641]; G1 to G0 transition [GO:0070314]; gamma-aminobutyric acid metabolic process [GO:0009448]; glial cell development [GO:0021782]; glutamine metabolic process [GO:0006541]; glycine metabolic process [GO:0006544]; L-serine biosynthetic process [GO:0006564]; neural tube development [GO:0021915]; neuron projection development [GO:0031175]; phosphoglycerate dehydrogenase activity [GO:0004617]; regulation of gene expression [GO:0010468]; small molecule metabolic process [GO:0044281]; spinal cord development [GO:0021510]; taurine metabolic process [GO:0019530]; threonine metabolic process [GO:0006566]	electron carrier activity [GO:0009055]; NAD binding [GO:0051287]; phosphoglycerate dehydrogenase activity [GO:0004617]	cytosol [GO:0005829]; extracellular vesicular exosome [GO:0070062]
A8MUB1	Tubulin alpha-4A chain OS=Homo sapiens GN=TUBA4A PE=2 SV=1 - [A8MUB1_HUMAN]	1		1	2	1		1	3	4.16%		2.31%	6.24%	2.16		2.89	6.95			
B7Z1G4	cDNA FLJ61601, highly similar to Inosine-5'-monophosphate dehydrogenase 2 (EC 1.1.1.205) OS=Homo sapiens PE=2 SV=1 - [B7Z1G4_HUMAN]	1	1	1	2	1	1	1	2	3.08%	3.08%	3.08%	7.00%	2.43	2.02	2.41	4.74	oxidoreductase activity [GO:0016491]	oxidoreductase activity [GO:0016491]	
P13929	Isoform 3 of Beta-enolase OS=Homo sapiens GN=ENO3 - [ENOB_HUMAN]	2		2	1	2		2	2	8.44%		8.44%	4.60%	5.36		4.85	4.81	aging [GO:0007568]; carbohydrate metabolic process [GO:0005975]; gluconeogenesis [GO:0006094]; glucose metabolic process [GO:0006006]; glycolytic process [GO:0006096]; pathogenesis [GO:0009405]; phosphopyruvate hydratase activity [GO:0004634]; response to drug [GO:0042493]; skeletal muscle tissue regeneration [GO:0043403]; small molecule metabolic process [GO:0044281]	magnesium ion binding [GO:0000287]; phosphopyruvate hydratase activity [GO:0004634]	cytoplasm [GO:0005737]; cytosol [GO:0005829]; extracellular space [GO:0005615]; extracellular vesicular exosome [GO:0070062]; phosphopyruvate hydratase complex [GO:0000015]; plasma membrane [GO:0005886]
Q9UK31	Pyruvate kinase M2 (Fragment) OS=Homo sapiens PE=4 SV=1 - [Q9UK31_HUMAN]	1	1	1	1	1	1	1	1	39.29%	39.29%	39.29%	39.29%	2.34	3.06	2.67	2.8	glycolytic process [GO:0006096]; pyruvate kinase activity [GO:0004743]	magnesium ion binding [GO:0000287]; potassium ion binding [GO:0030955]; pyruvate kinase activity [GO:0004743]	
B4DVQ0	cDNA FLJ58286, highly similar to Actin, cytoplasmic 2 OS=Homo sapiens PE=2 SV=1 - [B4DVQ0_HUMAN]	2		1	2	2		1	3	11.11%		3.00%	10.21%	5.19		2.61	6.46		ATP binding [GO:0005524]	
B3KML9	cDNA FLJ11352 fis, clone HEMBA1000020, highly similar to Tubulin beta-2C chain OS=Homo sapiens PE=2 SV=1 - [B3KML9_HUMAN]	5		4		5		4		13.35%		10.83%		12.3		8.44		GTPase activity [GO:0003924]; microtubule-based process [GO:0007017]; protein polymerization [GO:0051258]	GTPase activity [GO:0003924]; GTP binding [GO:0005525]; structural constituent of cytoskeleton [GO:0005200]	microtubule [GO:0005874]
Q5T8M8	Actin, alpha skeletal muscle OS=Homo sapiens GN=ACTA1 PE=2 SV=1 - [Q5T8M8_HUMAN]	2		1	1	2		1	2	13.59%		3.48%	5.57%	5.37		2.61	4.14			
A8K854	HCG1983504, isoform CRA_f OS=Homo sapiens GN=TUBB3 PE=2 SV=1 - [A8K854_HUMAN]	3		2		3		2		8.47%		5.82%		7.14		4.26				
H0YB39	Heterogeneous nuclear ribonucleoprotein H (Fragment) OS=Homo sapiens GN=HNRNPH1 PE=4 SV=1 - [H0YB39_HUMAN]	2	1	1	1	2	1	1	2	12.27%	6.14%	6.14%	6.14%	5.29	2.2	3.37	5.65		nucleic acid binding [GO:0003676]; nucleotide binding [GO:0000166]	
K7EK77	ATP synthase subunit alpha, mitochondrial (Fragment) OS=Homo sapiens GN=ATP5A1 PE=2 SV=1 - [K7EK77_HUMAN]	2		1		2		1		13.04%		5.80%		4.48		2.24		ATP metabolic process [GO:0046034]; lipid metabolic process [GO:0006629]; proton-transporting ATP synthase activity, rotational mechanism [GO:0046933]	ATP binding [GO:0005524]; proton-transporting ATP synthase activity, rotational mechanism [GO:0046933]	mitochondrial inner membrane [GO:0005743]
P06733	Alpha-enolase OS=Homo sapiens GN=ENO1 PE=1 SV=2 - [ENOA_HUMAN]	3		1	1	3		1	2	13.13%		4.15%	4.15%	8.41		2.23	4.81	carbohydrate metabolic process [GO:0005975]; gluconeogenesis [GO:0006094]; glucose metabolic process [GO:0006006]; glycolytic process [GO:0006096]; negative regulation of cell growth [GO:0030308]; negative regulation of transcription, DNA-templated [GO:0045892]; negative regulation of transcription from RNA polymerase II promoter [GO:0000122]; pathogenesis [GO:0009405]; phosphopyruvate hydratase activity [GO:0004634]; response to virus [GO:0009615]; sequence-specific DNA binding transcription factor activity [GO:0003700]; small molecule metabolic process [GO:0044281]; transcription, DNA-templated [GO:0006351]; transcription corepressor activity [GO:0003714]	DNA binding [GO:0003677]; GTPase binding [GO:0051020]; magnesium ion binding [GO:0000287]; phosphopyruvate hydratase activity [GO:0004634]; poly(A) RNA binding [GO:0044822]; sequence-specific DNA binding transcription factor activity [GO:0003700]; transcription corepressor activity [GO:0003714]	cytoplasm [GO:0005737]; cytosol [GO:0005829]; extracellular space [GO:0005615]; extracellular vesicular exosome [GO:0070062]; M band [GO:0031430]; membrane [GO:0016020]; nucleus [GO:0005634]; phosphopyruvate hydratase complex [GO:0000015]; plasma membrane [GO:0005886]
B4DE77	cDNA FLJ55189, highly similar to Tubulin beta-4 chain OS=Homo sapiens PE=2 SV=1 - [B4DE77_HUMAN]	4	1	2		4	1	2		10.02%	4.16%	4.65%		9.82	2.35	4.33		GTPase activity [GO:0003924]; microtubule-based process [GO:0007017]; protein polymerization [GO:0051258]	GTPase activity [GO:0003924]; GTP binding [GO:0005525]; structural constituent of cytoskeleton [GO:0005200]	microtubule [GO:0005874]
Q9BUU9	TUBB protein (Fragment) OS=Homo sapiens GN=TUBB PE=2 SV=1 - [Q9BUU9_HUMAN]	7		4	1	7		4	1	25.00%		13.53%	4.41%	17.51		8.41	2.86	GTPase activity [GO:0003924]; protein polymerization [GO:0051258]; spindle assembly [GO:0051225]	GTPase activity [GO:0003924]; GTP binding [GO:0005525]; structural constituent of cytoskeleton [GO:0005200]	microtubule [GO:0005874]; nucleus [GO:0005634]
F6KPG5	Albumin (Fragment) OS=Homo sapiens PE=2 SV=1 - [F6KPG5_HUMAN]	7	1	1	1	12	1	1	1	13.16%	2.56%	2.56%	2.05%	38.5	2.94	2.95	2.37	transport [GO:0006810]	0	extracellular space [GO:0005615]
