# Supplementary material for: Mutation-specific non-canonical pathway of PTEN as a distinct therapeutic target for glioblastoma
Source: Cell Death Dis. 2021 Apr 7;12(4):374. doi: 10.1038/s41419-021-03657-0 (PMC8027895; doi:10.1038/s41419-021-03657-0)
Supplement: Supplementary file 15 — Supplementary table S2 [file 41419_2021_3657_MOESM15_ESM.pdf]

sample_ID	PTEN_MUT	mutation_type	ref	alt	ref_count	alt_count	VAF	CDS_mutation	AA_mutation
P001	PTEN	frameshift_variant	GT	G	198	4	0.01980198	c.166delT	p.L57Wfs*42
P002	PTEN	missense_variant	A	G	34	148	0.813186813	c.65A>G	p.D22G
P003	PTEN	Substitution - Nonsense	C	A	29	127	0.814102564	c.176C>A	p.S59*
P004	PTEN	stop_gained	G	A	112	23	0.17037037	c.822G>A	p.W274*
P005	PTEN	frameshift_variant	C	CT	32	196	0.859649123	c.956_957insT	p.L320Ffs*5
P006	PTEN	Substitution - Nonsense	C	G	419	23	0.052036199	c.720C>G	p.Y240*
P007	PTEN	inframe_deletion	AGAT	A	540	57	0.095477387	c.591_593delGAT	p.M198del
P008	PTEN	Substitution - Missense	G	A	68	275	0.801749271	c.389G>A	p.R130Q
P009	PTEN	frameshift_variant	GAGAT	G	47	259	0.846405229	c.43_46delAGAT	p.R15Ifs*8
P010	PTEN	Substitution - Missense	G	A	241	146	0.377260982	c.389G>A	p.R130Q
P011	PTEN	inframe_deletion	GTTACCT	G	29	164	0.849740933	c.739_744delTTACCT	p.L247_P248delinsdel
P012	PTEN	Substitution - Nonsense	C	T	147	247	0.626903553	c.733C>T	p.Q245*
P013	PTEN	splice_donor_variant	T	A	14	151	0.915151515	c.801+2T>A	NA
P014	PTEN	missense_variant	G	T	104	33	0.240875912	c.212G>T	p.C71F
P015	PTEN	Substitution - Missense	G	A	41	385	0.903755869	c.386G>A	p.G129E
P016	PTEN	Substitution - Nonsense	C	T	33	198	0.857142857	c.781C>T	p.Q261*
P017	PTEN	missense_variant	G	A	68	196	0.742424242	c.140G>A	p.R47K
P018	PTEN	Substitution - Nonsense	C	T	197	40	0.168776371	c.511C>T	p.Q171*
P019	PTEN	Substitution - Nonsense	T	G	216	174	0.446153846	c.48T>G	p.Y16*
P020	PTEN	missense_variant	A	C	294	150	0.337837838	c.353A>C	p.H118P
P021	PTEN	Substitution - Nonsense	C	T	165	36	0.179104478	c.1003C>T	p.R335*
P022	PTEN	Substitution - Nonsense	C	A	54	93	0.632653061	c.195C>A	p.Y65*
P023	PTEN	frameshift_variant	G	T	194	152	0.439306358	c.997G>T	p.A333S
P024	PTEN	frameshift_variant	CT	C	8	298	0.973856209	c.884delT	p.L295Hfs*12
P025	PTEN	frameshift_variant	T	TA	272	153	0.36	c.740_741insA	p.P248Tfs*5
P026	PTEN	inframe_deletion	ATAT	A	47	226	0.827838828	c.147_149delTAT	p.I50del
P027	PTEN	splice_donor_variant	CTG	C	22	12	0.352941176	c.209_209+1delTG	NA
P028	PTEN	Substitution - Missense	A	G	51	82	0.616541353	c.182A>G	p.H61R
P029	PTEN	Substitution - Nonsense	C	T	30	144	0.827586207	c.697C>T	p.R233*
P030	PTEN	Substitution - Missense	T	C	148	57	0.27804878	c.202T>C	p.Y68H
P031	PTEN	frameshift_variant	AT	A	172	474	0.73374613	c.95delT	p.I33Lfs*21
P032	PTEN	Substitution - Missense	C	T	41	620	0.937972769	c.367C>T	p.H123Y
P033	PTEN	missense_variant	A	G	35	15	0.3	c.778A>G	p.K260E
P034	PTEN	Substitution - Missense	T	C	133	336	0.71641791	c.644T>C	p.F215S
P035	PTEN	missense_variant	T	G	454	272	0.374655647	c.312T>G	p.F104L
P036	PTEN	Unknown	G	A	11	16	0.592592593	c.164+1G>A	NA
P037	PTEN	frameshift_variant	ACTTT	A	41	89	0.684615385	c.956_959delCTTT	p.T319Kfs*24
P038	PTEN	Substitution - Missense	T	C	311	17	0.051829268	c.406T>C	p.C136R
P039	PTEN	frameshift_variant	TCG	T	335	172	0.339250493	c.424_425delCG	p.R142Gfs*37
P040	PTEN	missense_variant	C	A	158	205	0.564738292	c.506C>A	p.P169H
P041	PTEN	frameshift_variant	GTACT	G	75	219	0.744897959	c.950_953delTACT	p.T319*
P042	PTEN	Substitution - Missense	T	C	42	62	0.596153846	c.406T>C	p.C136R
P043	PTEN	Substitution - Missense	T	C	144	100	0.409836066	c.406T>C	p.C136R
P044	PTEN	Substitution - Missense	C	T	74	4	0.051282051	c.500C>T	p.T167I
P045	PTEN	Substitution - Missense	G	A	38	28	0.424242424	c.389G>A	p.R130Q
P046	PTEN	inframe_deletion	AGAT	A	30	28	0.482758621	c.591_593delGAT	p.M198del
P047	PTEN	frameshift_variant	T	TA	4	38	0.904761905	c.545_546insA	p.N184Efs*6
P048	#N/A	splice_donor_variant	G	A	25	24	0.489795918	c.1026+1G>A	NA
P049	PTEN	frameshift_variant	GA	G	173	11	0.059782609	c.941delA	p.E314Dfs*3
P050	PTEN	Substitution - Missense	G	A	238	50	0.173611111	c.394G>A	p.G132S
P051	PTEN	frameshift_variant	GTACT	G	89	122	0.578199052	c.950_953delTACT	p.T319*
P052	PTEN	Substitution - Missense	T	C	614	446	0.420754717	c.401T>C	p.M134T
P053	PTEN	inframe_deletion	TGTA	T	29	20	0.408163265	c.157_159delGTA	p.V53del
P054	PTEN	Substitution - Missense	G	A	150	56	0.27184466	c.494G>A	p.G165E
P055	PTEN	Substitution - Missense	G	A	34	103	0.751824818	c.212G>A	p.C71Y
P056	PTEN	inframe_deletion	GTAT	G	416	537	0.563483736	c.526_528delTAT	p.Y176del
P057	PTEN	Substitution - Missense	G	T	362	130	0.264227642	c.402G>T	p.M134I
P058	PTEN	missense_variant	C	T	137	13	0.086666667	c.1076C>T	p.A359V
P059	PTEN	Substitution - Missense	T	C	1056	124	0.105084746	c.401T>C	p.M134T
P060	PTEN	Substitution - Missense	A	G	7	32	0.820512821	c.530A>G	p.Y177C

P061	PTEN	inframe_deletion	GTAT	G	19	13	0.40625	c.526_528delTAT	p.Y176del
P062	PTEN	missense_variant	G	C	82	110	0.572916667	c.493G>C	p.G165R
P063	PTEN	frameshift_variant	GT	G	26	13	0.333333333	c.166delT	p.L57Wfs*42
P064	PTEN	Substitution - Nonsense	C	T	44	50	0.531914894	c.1003C>T	p.R335*
P065	PTEN	Substitution - Missense	G	A	119	124	0.510288066	c.394G>A	p.G132S
P066	PTEN	Substitution - Missense	C	T	84	88	0.511627907	c.737C>T	p.P246L
P067	PTEN	Substitution - Nonsense	C	G	21	31	0.596153846	c.540C>G	p.Y180*
P068	PTEN	missense_variant	C	T	23	84	0.785046729	c.1076C>T	p.A359V
P069	PTEN	frameshift_variant	GTACT	G	29	79	0.731481481	c.950_953delTACT	p.T319*
P070	PTEN	Substitution - Nonsense	G	A	33	3	0.083333333	c.822G>A	p.W274*
P071	PTEN	frameshift_variant	GA	G	28	104	0.787878788	c.896delA	p.I300Sfs*7
P072	PTEN	missense_variant	G	C	8	16	0.666666667	c.493G>C	p.G165R
P073	PTEN	frameshift_variant	GT	G	114	4	0.033898305	c.166delT	p.L57Wfs*42
P074	PTEN	Substitution - Missense	G	T	9	43	0.826923077	c.1026G>T	p.K342N
P075	PTEN	Substitution - Missense	C	T	33	29	0.467741935	c.830C>T	p.T277I
P076	PTEN	missense_variant	C	G	34	12	0.260869565	c.610C>G	p.P204A
P077	PTEN	missense_variant	C	G	7	165	0.959302326	c.506C>G	p.P169R
P078	PTEN	frameshift_variant	TA	T	56	32	0.363636364	c.988delA	p.D331Tfs*13
P079	PTEN	Substitution - Missense	G	C	48	94	0.661971831	c.256G>C	p.A86P
P080	PTEN	frameshift_variant	TA	T	54	6	0.1	c.795delA	p.K267Rfs*9
P081	PTEN	frameshift_variant	TA	T	33	29	0.467741935	c.988delA	p.D331Tfs*13
P082	PTEN	frameshift_variant	TA	T	41	49	0.544444444	c.988delA	p.D331Tfs*13
P083	PTEN	Substitution - Missense	C	T	11	65	0.855263158	c.737C>T	p.P246L
P084	PTEN	splice_donor_variant	G	T	16	14	0.466666667	c.209+1G>T	NA
P085	PTEN	Substitution - Missense	T	C	69	15	0.178571429	c.406T>C	p.C136R
P086	PTEN	missense_variant	G	T	72	83	0.535483871	c.402G>T	p.M134I
P087	PTEN	Substitution - Missense	C	T	95	70	0.424242424	c.277C>T	p.H93Y
P088	PTEN	missense_variant	T	C	16	6	0.272727273	c.497T>C	p.V166A
