# Supplementary material for: Mutation-specific non-canonical pathway of PTEN as a distinct therapeutic target for glioblastoma
Source: Cell Death Dis. 2021 Apr 7;12(4):374. doi: 10.1038/s41419-021-03657-0 (PMC8027895; doi:10.1038/s41419-021-03657-0)
Supplement: Supplementary file 16 — Supplementary table S3 [file 41419_2021_3657_MOESM16_ESM.docx]

**Primers for mutagenesis**

| Mutation | Primer | Sequence |
| --- | --- | --- |
| D24N | FW | GAGGATGGATTCGACTTAAACTTGACCTATATTTATC |
|  | RV | TTGATATCTCCTTTTGTTTCTGCTAACGATC |
| H93Y | FW | CAATATCCTTTTGAAGACTATAACCCACCACAGCTAG |
|  | RV | TGCAACTCTGCAATTAAATTTGGCGGTG |
| R130Q | FW | GTAAAGCTGGAAAGGGACAAACTGGTGTAATGATATG |
|  | RV | AGTGAATTGCTGCAACATGATTGTCATCTTC |
| G132D | FW | GGAAAGGGACGAACTGATGTAATGATATGTGC |
|  | RV | AGCTTTACAGTGAATTGCTGCAACATG |
| R173C | FW | GGAGTAACTATTCCCAGTCAGAGGTGCTATGTGTATTATTATAGCTACC |
|  | RV | CTTTTTGTCTCTGGTCCTTACTTCCCCATAG |
| K289E | FW | CAGAGGAAACCTCAGAAGAAGTAGAAAATGGAAG |
|  | RV | GTCCTGGTATGAAGAATGTATTTACCCAAAAG |
